# Pharmacophore-Based Virtual Screening and In-Silico Explorations of Biomolecules (Curcumin Derivatives) of *Curcuma longa* as Potential Lead Inhibitors of ERBB and VEGFR-2 for the Treatment of Colorectal Cancer

**DOI:** 10.3390/molecules28104044

**Published:** 2023-05-12

**Authors:** Syeda Abida Ejaz, Mubashir Aziz, Mohamed Fawzy Ramadan, Ammara Fayyaz, Muhammad Sajjad Bilal

**Affiliations:** 1 Department of Pharmaceutical Chemistry, Faculty of Pharmacy, The Islamia University of Bahawalpur, Bahawalpur 63100, Pakistan; 2 Department of Clinical Nutrition, Faculty of Applied Medical Sciences, Umm Al-Qura University, Makkah 21955, Saudi Arabia

**Keywords:** pharmacophore, VEGFR, DFTs, molecular docking, molecular dynamic simulations

## Abstract

The newly FDA-approved drug, Axitinib, is an effective therapy against RTKs, but it possesses severe adverse effects like hypertension, stomatitis, and dose-dependent toxicity. In order to ameliorate Axitinib’s downsides, the current study is expedited to search for energetically stable and optimized pharmacophore features of 14 curcumin (1,7-bis(4-hydroxy-3-methoxyphenyl)hepta-1,6-diene-3,5-dione) derivatives. The rationale behind the selection of curcumin derivatives is their reported anti-angiogenic and anti-cancer properties. Furthermore, they possessed a low molecular weight and a low toxicity profile. In the current investigation, the pharmacophore model-based drug design, facilitates the filtering of curcumin derivatives as VEGFR2 interfacial inhibitors. Initially, the Axitinib scaffold was used to build a pharmacophore query model against which curcumin derivatives were screened. Then, top hits from pharmacophore virtual screening were subjected to in-depth computational studies such as molecular docking, density functional theory (DFT) studies, molecular dynamics (MD) simulations, and ADMET property prediction. The findings of the current investigation revealed the substantial chemical reactivity of the compounds. Specifically, compounds **S8**, **S11**, and **S14** produced potential molecular interactions against all four selected protein kinases. Docking scores of −41.48 and −29.88 kJ/mol for compounds **S8** against VEGFR1 and VEGFR3, respectively, were excellent. Whereas compounds **S11** and **S14** demonstrated the highest inhibitory potential against ERBB and VEGFR2, with docking scores of −37.92 and −38.5 kJ/mol against ERBB and −41.2 and −46.5 kJ/mol against VEGFR-2, respectively. The results of the molecular docking studies were further correlated with the molecular dynamics simulation studies. Moreover, HYDE energy was calculated through SeeSAR analysis, and the safety profile of the compounds was predicted through ADME studies.

## 1. Introduction

Curcumin, the biomolecule obtained from turmeric (*Curcuma longa*, 1.5–3% wt.), has pleiotropic properties, including chemo-sensitizing, anti-oxidant, chemo-protective, anti-inflammatory, anti-proliferative, hepato-protective, anti-metastatic, and anti-cancer properties. Curcumin affects most signaling pathways due to its complicated chemistry and molecular structure. Any imbalance in signaling pathways may lead to metastasis [1]. Among the most common types of cancer, colorectal cancer (CRC) is one of the leading cancers, accounting for approximately 10% of cancer incidence and mortality in both males and females [2]. Every year, numerous people are diagnosed with, and die of, colorectal cancer; by 2014, the number of people who died after being diagnosed with cancer had reached 14.5 million, and the number will be expected to increase to nearly 19 million by 2024 [3]. The CRC is either metastatic or locally advanced, and surgical resection is unlikely to be curative. For most patients, chemotherapy can enhance survival and is the only mode of treatment [4,5,6].

There has been much interest in several novel therapeutic approaches for cancer treatment that target the molecular pathways that regulate tumor cell growth or survival. Potential anti-neoplastic treatment targets, such as epidermal growth factor receptor (EGF-R) and vascular endothelial growth factor receptor (VEGF-R), have been investigated [7]. EGF-R and VEGF-R are examples of receptor tyrosine kinases (RTKs), which are trans-membrane proteins with an extracellular ligand-binding domain and an intracellular tyrosine kinase catalytic domain. After binding to their catalytic site, most RTKs form dimers and undergo autophosphorylation of intracellular tyrosine residues [7,8]. Numerous cellular signaling pathways that promote cell growth, survival, and angiogenesis are triggered in response to RTK activation. The emergence of genetic makeup changes is a mediator in the development of colorectal or renal cancer disease. The accumulation of specific growth-inducing factors, such as hypoxia-inducing factors, is caused by mutation or gene silencing (HIF-alpha). These built-up substances function as transcriptional agents that move into the nucleus and trigger the synthesis of growth factors like platelet-derived and epithelial growth factors. These elements begin encouraging metastasis, cell growth, proliferation, and angiogenesis. It is also hypothesized that cancer cells circumvent usual growth constraints by inappropriately activating RTKs by mutation, overexpression, or ectopic ligand production, which is a typical feature of human tumor genesis and progression [9]. In light of this, RTK signal transduction control has emerged as a primary focus of oncology medication development, and several agents have been developed that primarily target the VEGFR signaling pathways.

Potential tyrosine kinase agents such as Axitinib, 5-fluorouracil (5-FU), irinotecan, and oxaliplatin possessed potential inhibition activity against VEGFR 1–3. Other agents that operate by blocking the tyrosine kinase domain of epidermal growth factor receptor (EGFR) utilizing monoclonal antibodies, such as cetuximab or panitumumab, are also available for the treatment of CRC [10,11]. Similarly, drugs that block VEGF receptor activation prevent the induction of metastasis. Several anti-angiogenic agents that target VEGF, including mAbs, TKIs, and decoy compounds (e.g., VEGF Trap), have been entered into clinical practice or are under clinical investigation [12]. Among all these tyrosine agents, only Axitinib is the latest FDA-approved drug integrated into international therapeutic guidelines for treating VEGFR-associated malignancies [13]. Axitinib is an indole derivative that has demonstrated potent and selective activity against multiple cancer cell lines, including renal, colorectal, thyroid, and non-small cell lung cancer disease [14]. Axitinib competitively binds to the ATP binding site of tyrosine kinase and inhibits phosphorylation [15]. In addition, it was reported that Axitinib blocked several growth factors in nano molar ranges, including platelet-derived growth factors, but it remained more selective toward RTKs [15]. However, the use of Axitinib and other kinase inhibitors is associated with certain disadvantages, such as the development of tolerance [16], toxicity, pharmacokinetic instability, and side effects. In particular, Axitinib developed dose-limiting toxicities (stomatitis and hypertension) and severe adverse effects such as myalgia, fatigue, gout, diarrhea, and hypertension [17].

Moreover, cross-tolerance and combination therapy trial data are insufficient to support the treatment therapy’s safety in many individuals [17]. Therefore, there is a strong rationale for designing selective inhibitors of both targets to eradicate cancer with the least resistance and side effects. Furthermore, these findings encourage us to develop alternative scaffolds for treating RTKs associated with cancer malignancies [11,12,18]. Among the various types of natural analogues, curcumin derivatives are considered important pharmaceutical agents, possessing anti-angiogenic and anticancer properties [19]. In addition, they are considered promising chemotherapeutic treatment strategies due to their low molecular weight and lack of toxicity against normal cells [20].

Furthermore, they have been reported for their role in growth suppression and apoptosis induction in various cancer cell lines (in vitro), i.e., inhibition of vascular endothelial cell (VEC) proliferation. Moreover, their anti-tumor capabilities have also been identified via in-vivo approaches, i.e., in vivo capillary tube formation and growth [21]. Based on these properties, curcumin analogues remain the lead molecules for the design of analogs with similar safety profiles, increased activity, and better pharmacokinetic profiles [22].

The current study aims to evaluate the curcumin derivatives as the inhibitors of ERBB, VEGFR1, VEGFR3 and VEGFR2 using various in silico approaches. The current study has utilized Axitinib as a parent scaffold for generating a pharmacophore query model against which a library of curcumin derivatives was screened via pharmacophore-based virtual screening, which could generate energetically optimized pharmacophores for lead discovery. The top-ranked hits retrieved via pharmacophore-based virtual screening were further subjected to advanced in-silico approaches. Initially, DFT calculations were performed to understand the electronic properties of all compounds, and optimized structures were obtained for molecular docking studies. Energy-based docking studies were then used to determine the ligand’s approximate/plausible positions within the receptor active site and the binding affinities. In addition to the docking studies, molecular dynamic simulation was performed to identify the stability of the docked complex.

**Figure 1:**
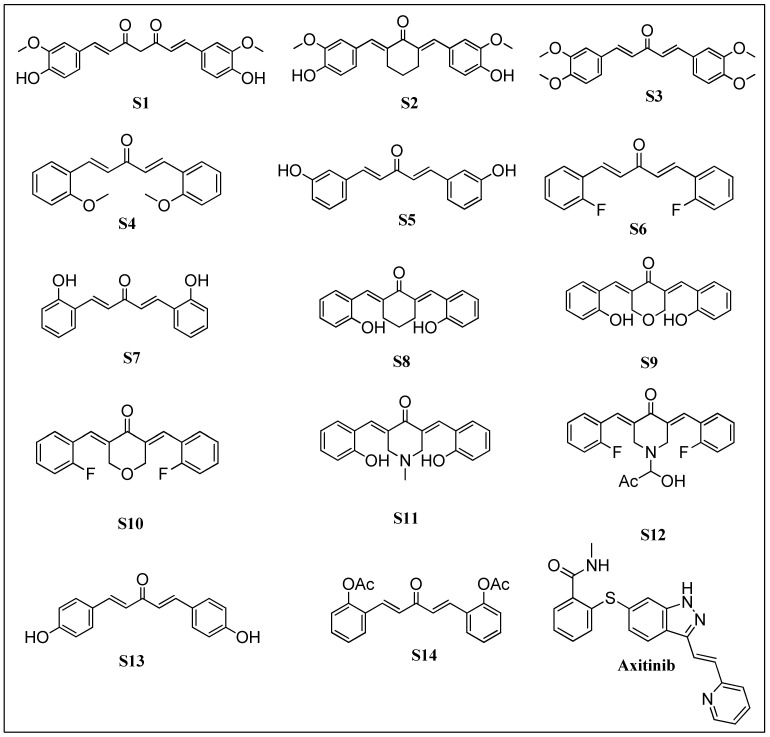
Curcumin analogues and FDA-approved Axitinib [19].

Moreover, a similarity search was performed for Axitinib and curcumin derivatives using Tanimoto and Dice similarity coefficients. The results will serve as a new direction for analyzing curcumin derivatives for treating RTKs associated cancer malignancies. Figure 1 depicts the curcumin analogues and Axitinib.

## 2. Results and Discussion

### 2.1. Preparation of Chemical Database

The curcumin derivatives were selected on the basis of their broad range of biological activities. The curcumin derivatives were previously reported as potential anticancer agents against various cancer cell lines, including melanoma RPMI 7951, human breast cancer, MDA-MB-231, and human umbilical vein endothelial cells, HUVEC [15]. A total of 14 curcumin derivatives were screened against multiple cell lines, and the chemical structures of each derivative were retrieved from the PubChem database. All these derivatives were retrieved in SDF format from the PubChem database and subjected to a preliminary energy minimization process before being converted to the desired format for further in-silico investigations. The IUPAC naming of all retrieved curcumin derivatives is provided in Table 1.

### 2.2. Generation of Pharmacophore Model

**Figure 2:**
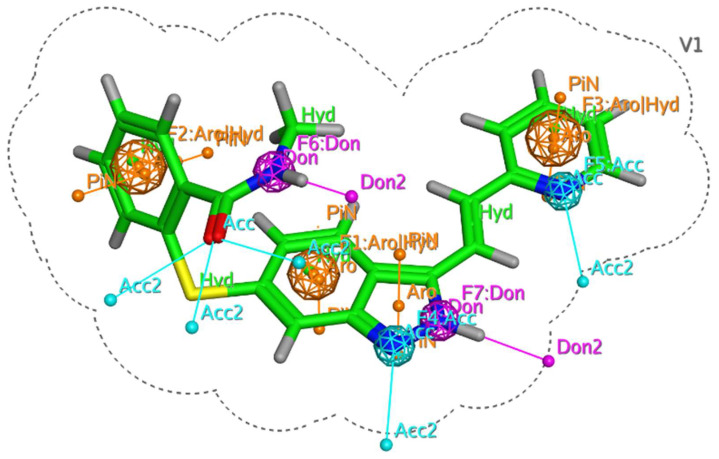
Generated chemical features of Axitinib based on intermolecular interactions.

The single protein–ligand complex can be used to define chemical features based on intermolecular interactions observed with the complex. In the present study, VEGFR-2 in complex with standard Axitinib was retrieved from the Protein Data Bank (PDB ID 4AG8) and subjected to pharmacophore model building. The interactions produced by Axitinib laid the foundation for the generation of pharmacophore features. The database consisting of 14 curcumin derivatives was screened against generated features, and the best-fitted compound was prioritized as a hit molecule. Based on intermolecular interactions, a total of seven features were generated, i.e., two hydrogen bond acceptors (blue sphere), two hydrogen bond donors (purple sphere), and three hydrophobic (orange spheres), as shown in Figure 2. In addition, four hydrogen bond features were observed, i.e., two hydrogen bond donor producing interactions with GLU885 and GLU917 and two hydrogen bond acceptor features involving CYS919 and LEU840 residues in bonding.

### 2.3. Pharmacophore-Based Virtual Screening

**Figure 3:**
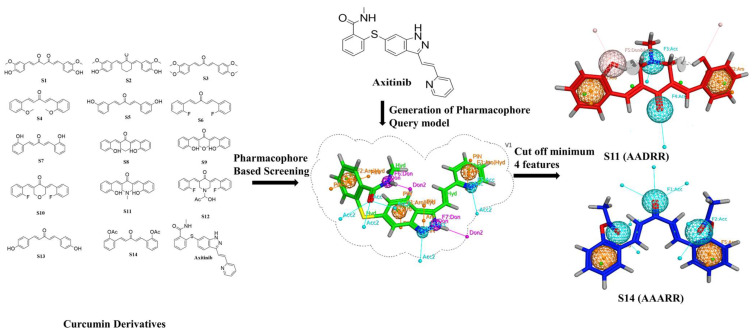
An Illustration of the pharmacophore-based virtual screening workflow.

After the generation of a pharmacophore query model, the curcumin database was screened against Axitinib’s predefined chemical features. It was observed that compounds **S11** and **S14** showed the best-fit chemical features. The compound **S11** showed five pharmacophore features AADRR (one donor, two acceptors, and two aromatics) with an RMSD value of 0.54 angstrom. Similarly, another best-fit compound, **S14**, showed five chemical features AAARR (three donors and two aromatics) with RMSD values less than 0.9 angstroms. Both these compounds involved important molecular interactions with amino acid residues at the active site. Figure 3 illustrates the generation of the pharmacophore query model on the basis of molecular interactions between Axitinib and VEGFR2. A total of seven pharmacophoric features were generated, against which compound **S11** and **S14** were found to be the best matches both with five features. For each compound the cut off value was set to a minimum of four. Any compound with less than four pharmacophoric features was omitted from the hit candidates. The generated and matched chemical features are shown in Figure 3.

### 2.4. Similarity Index

Implementing the similarity principle is essential for evaluating a query compound’s biological and chemical properties and a target dataset. In the present study, Axitinib was utilized as a query molecule, and top hits obtained from pharmacophore-based virtual screening were considered the test dataset. Initially, MACCS and Morgan fingerprints [23] were generated for each molecule in the query and test dataset. Afterward, two similarity coefficients, i.e., Tanimoto and Dice coefficients, were applied using the open-source RDKIT library on both generated fingerprints. The rationale behind generating two different types of fingerprints and implementing similarity coefficients was to enhance the reliability and accuracy of generated outputs. As a result, it was observed that compound **S11** showed a slightly higher similarity index with Axitinib, whereas **S14** was slightly lower in similarity index. The exact values are given in Table 2.

### 2.5. Density Function Theory (DFTs)

The structural geometries of curcumin derivatives were optimized to steepest decent gradient and frequency calculations were performed using DFT/B3LYP functional correlation and 3-21G as a basis set. In order to perform DFT calculations of curcumin derivatives, all the structure files were converted to the desired format using the Gauss View 6 program after specifying the calculation parameters. All compounds’ geometry was optimized in the gas phase.

The dipole moment and optimization energy of all candidate compounds were determined to understand the extent of reactivity and stability. Additional descriptors such as electronegativity (χ = −1/2(ELUMO + EHOMO), chemical hardness (η = 1/2(ELUMO − EHOMO), softness (S = 1/2η), electron donating power (ω− = (3I + A)2/16(I − A)), electron accepting power (ω+ = (I + 3A)2/16(I − A)), and electrophilicity index (ω = µ/2η) were determined using ionization potential and electron affinity values. The various descriptor values, dipole moment, and optimization energies for all the compounds are given in Table 3.

The hardness of any compound is associated with its ability to react with molecules in its vicinity. Therefore, any molecule with a high hardness value is considered the least reactive and more stable. The density functional theory calculations were performed for all the compounds, and according to the results, compound **S5** showed the highest hardness value, making it resistant to being attacked by other molecules. In the same way, **S2** was found to be the most reactive because of it had the lowest hardness value of **S2**. As the electronegativity of a compound is its ability to accept an electron from the environment, the DFT results indicated that all compounds showed almost similar electronegativity, but **S5** was found to be more prone to ionization from the environment and showed a slightly high electronegativity. The electrophilicity index of all compounds was also calculated, showing derivative **S5** to be the most electron-loving among all the compounds. The value of all the compounds for these descriptors is given in Table 4.

The results of frontier molecular orbitals energy, i.e., EHOMO and ELUMO, and their energy gap (ELUMO-EHOMO) also indicated that most of the compounds showed equal energy difference and were found to be stable. The values are given in Table 4. The results of other reactivity descriptors, i.e., electron-donating power and electron-accepting power, indicated that the extent of reactivity was also consistent with the results of other global reactivity descriptors. The value for all the compounds is given in Table 5.

**Figure 4:**
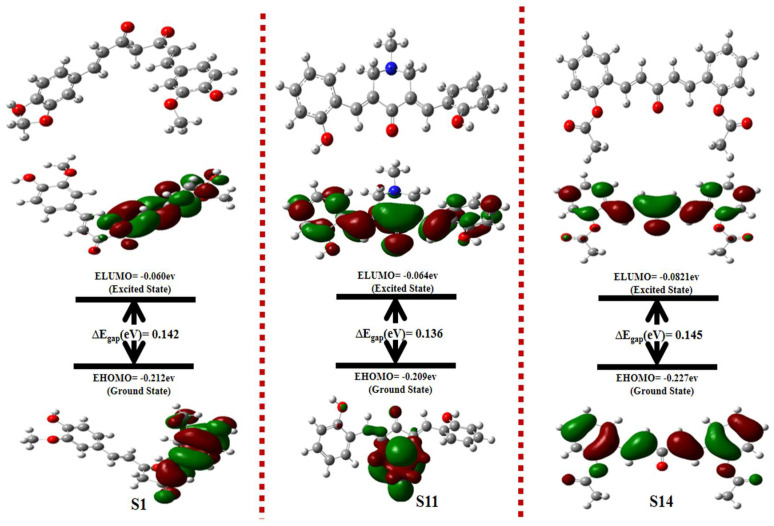
Optimized structures along with LUMO and HOMO energy transitions for **S1**, **S11**, and **S14**.

Ionization energy, along with the electron affinity of compounds, is another approach to understanding the stability and reactivity of a compound. The compounds with higher ionization energy values are least prone to lose electrons and have greater stability. It is also evident from the results that **S14** has the highest value of ionization energy which speaks to its inert nature and reliable stability. The same HOMO and LUMO energy, along with energy gap and optimization energy, is given in the tables for all the compounds. For example, from the results of DFT studies, the optimized structure, HOMO, and LUMO, along with their respective energy gap of the highly potent compounds, i.e., **S1**, **S11**, and **S14**, is given in Figure 4. It was notable that HOMO orbitals were localized around the phenyl ring of compound **S1**, whereas LUMO orbitals were delocalized around the acetate part of the compound. The energy gap between LUMO and HOMO orbitals was 0.142 eV for **S1**. In terms of compound **S11**, the HOMO orbitals were localized around the piperidine moiety, representing the electron-donating behavior of the piperidine moiety of the compound. The LUMO orbitals, on the other hand, were delocalized over the majority of the compound. The LUMO/HOMO energy gap for compound **S11** was at a minimum of 0.136 eV, representing the high chemical reactivity of the compound. The FMOs analysis of compound **S14** revealed that the whole compound was involved in electron-accepting and electron-donating properties, which corresponds to its high chemical reactivity profile.

### 2.6. Filtration for Drug-Likeness and Virtual Screening

The calculated pharmacokinetics of all the compounds showed that Lipinski’s rule of five (RO5); which represents the drug-likeness of the chemical, is not violated by any derivative. Due to appropriate water solubility, lipophilicity and permeability, almost all of the compounds showed excellent absorption. The high bioavailability of the compounds was confirmed by the number of rotatable bonds and polar surface area. The compounds’ toxicity profiles were also investigated. According to the projected results, all derivatives are non-carcinogenic and have no influence on immunotoxicity, mutagenicity, or cytotoxicity. The ADMET (absorption, distribution, metabolism, excretion, and toxicity) properties of the most powerful derivative were calculated to determine its appropriateness as a therapeutic molecule. The physicochemical properties were molecular weight, density, number of hydrogen bond acceptors (nHA), number of hydrogen bond donors (nHD), topological polar surface area (TPSA), log of aqueous solubility (LogS), log of the octanol–water partition coefficient (LogP), and logP at physiological concentrations (LogD) (Table 6).

Hydrogen bonding is an important chemical parameter in the determination of thermodynamic properties of a compound. Total Polar Surface Area (TPSA) has a significant role in the estimation of polarity which is a major factor contributing toward penetration and permeation. According to the ADMET profile, the compound **S1** showed the highest value of TPSA i.e., 93.6. Another parameter i.e., Log S, if a compound is sufficiently lipophilic and it has an ineffective range of aqueous solubility (Log S) then its permeation through membranes will be hindered, as is the case with **S6** which was found to be −6.046. The value of all other compounds is given in Table 4. The molecular weights of all derivatives lie within the optimal range (<500), except **S12** and in the same way nHA, nHD and TPSA values of all compounds were found to be within the permitted range, whereas derivative **S6** has minimum total polar surface area and **S2** has maximum polar surface area.

The compounds **S2**, **S9** and **S11** have acceptable values (−1 to −5.6) of log S with good aqueous solubility, while remaining derivatives showed deviation from the reference values. The compound **S2** exhibited an acceptable log *p* value while other compounds were found to be borderline with slightly higher values of log P. The log D value of all derivatives is found in-correlation with log p results as given in Table 7.

The absorption and distribution profile of all the compounds showed efficient HIA, COCA 2 permeability and MDCK Permeability which represented their potential to penetrate/permeate through cell membranes. Except **S11**, all the derivatives had efficient potential to cross BBB and showed CNS effects. As far as its interaction with P glycoprotein was concerned, excluding **S3**, all other derivatives were found to have good PGP substrate properties while **S1**, **S5** and **S7** derivatives showed maximum PGP inhibitions whereas derivative **S11** also showed moderate PGP inhibition activity, which proved its capability to permeate as shown in Table 8.

The metabolism of any drug is an important parameter to understand its behavior in the body. All derivatives showed CYP inhibition activity of varying degrees. All compounds had a moderate rate of renal clearance while **S2**, **S5** and **S7** were present with relatively higher rates, and **S11** had the highest value of renal clearance shown in Table 9.

Any compound with a high level of toxicity cannot be used as a drug and in this regard assessment of mutagenic potential is crucial in the development of drug. The results of toxicity parameters indicated that **S9**, **S10**, **S11** and **S14** have excellent safety profiles in terms of mutagenicity, but **S8** was moderately mutagenic and all other derivatives were toxic. The compounds **S1**, **S2**, **S3**, **S6** and **S10** did not show any carcinogenic potential and were found to be safe. Their safety indicated their appropriateness for drug development as they do not pose a carcinogenic threat in humans. The compounds **S4**, **S5**, **S7** and **S8** were moderately carcinogenic. Only **S5** and **S7** derivatives were not corrosive to the eye, compounds **S4**, **S5**, **S6**, **S7**, **S8** and **S9** showed non-irritant behavior to the cornea, which justified their ocular safety. **S6** was found to be moderately eye corrosive, whereas **S1**, **S2** and **S3** were found to be moderately irritant. Moreover, **S1**, **S2**, **S5**, **S7** and **S11** did not have respiratory toxicity the rest were moderately toxic (Table 10).

Compounds **S1** and **S2** did not activate the androgen receptor while **S3**, **S4**, **S5**, **S7**, and **S8** derivatives showed moderate activity while others may activate androgen receptors. Only **S1** and **S11** possessed activity for the ligand binding domain (LBD), **S8** and **S9** had moderate potential while the rest of the derivatives had no activity. The compounds **S2**, **S6**, **S10** and **S11** showed moderate activity for estrogen receptors but the rest of the compounds had no activity at all, and none of the compounds under study showed any evidence of antioxidant potential.

### 2.7. Molecular Docking Discussion

#### 2.7.1. Binding Interactions of ERBB

The Molecular Operating Environment (MOE) and AutoDock 4.2 were used to investigate the binding interactions of selected compounds with targeted proteins i.e., ERBB, VEGFR1, VEGFR-2 and VEGFR3. The MOE software predicted poses that were reliable and validated on the basis of RMSD values between native poses and regenerated poses, which motivate us to incorporate binding energies and docking conformations obtained through the MOE software. Multiple protein kinases were selected in order to evaluate the inhibitory potential and selectivity of curcumin derivatives against multiple protein kinases. It was observed that compound **S14** had demonstrated the highest selectivity and inhibitory potential against VEGFR2 and ERBB whereas compound **S8** was effective against VEGFR1 and VEGFR3. The binding energies of curcumin derivatives against all four targeted proteins are provided in Table 10, whereas the predicted inhibitory constant value (ki) is provided in a Supplementary File (Table S1). In the main manuscript, the binding interactions analysis of top ranked conformations of curcumin derivatives against VEGRF2 and ERBB tyrosine kinase is elaborated, whereas binding interactions analysis of top ranked curcumin derivatives (**S8**) against VEGFR1 and VEGFR3 is provided in the Supplementary File (Figures S1–S10).

The docked conformation of curcumin derivatives exhibited potential molecular interactions against all targeted proteins. Briefly, Compound **S11** and **S14** were top ranked hits identified through molecular docking and MD simulations studies. The binding energies of curcumin derivatives were better than the standard drug irinotecan. Initially, irinotecan was docked with ERBB and the VEGFR2 protein. The following amino acid residues were involved in the formation of the complex with the standard drug (ERBB tyrosine kinase): LYS273, ASP833, VAL704, ARG819, and LEU777. The major binding interactions of the reference compound, i.e., irinotecan, with the targeted protein (ERBB tyrosine kinase), were comprised of strong hydrogen bonds. Hydrogen bond interactions were discovered between the carboxylate group attached to irinotecan’s bipiperidine ring and LYS723, ASP833. The pink amino acid residues were hydrophilic groups, while the green hydrophobic amino acid residue (VAL724) formed a Pi-sigma interaction.

**Figure 5:**
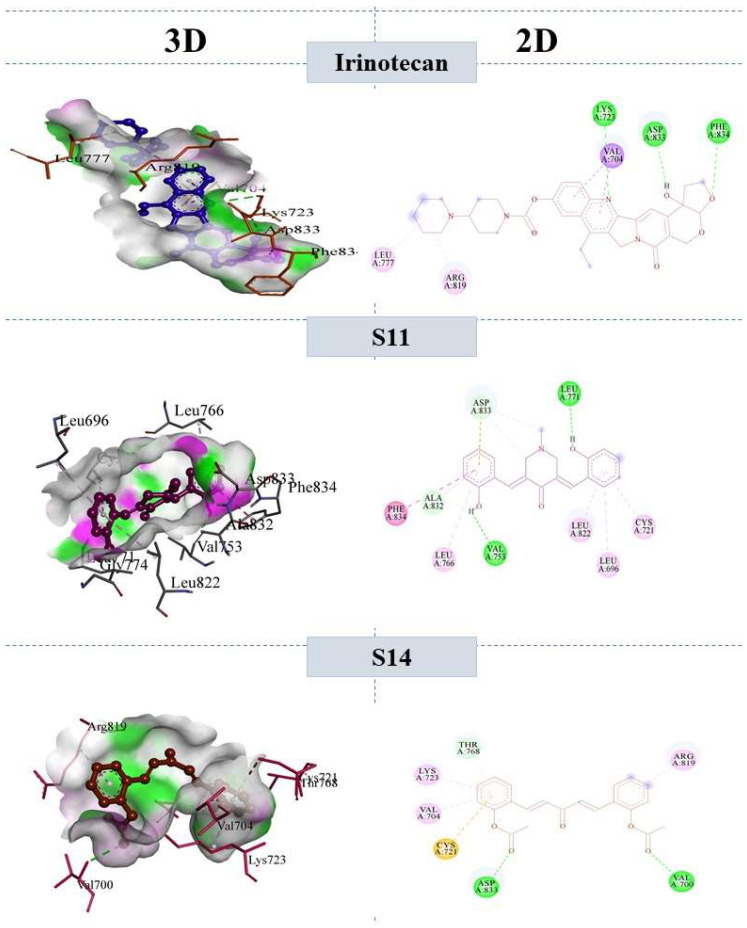
3D and 2D interaction of reference compound Irinotecan, **S11** and **S14** within the active pocket of ERBB tyrosine kinase.

The compounds **S11** and **S14** were involved in different molecular interactions with the following amino acid residues: PHE834, ALA832, ASP833, LEU771, LEU776, VAL753, LEU822, and LEU696, CYS721 for **S12**, and THR768, LYS723, VAL704, CYS721, ASP833, VAL700, and ARG819 for **S14**, respectively. The bonding and non-bonding interactions of **S11** and **S14** within the active pocket of the ERBB protein included the conventional hydrogen bond, the carbon–hydrogen bond, Van der Waals forces, and a weak pi-alkyl bond. The binding interactions revealed that the two strong hydrogen bonds were formed with VAL 753 and LEU 771. In addition, various carbon–hydrogen bonds were formed with ALA832, ASP833. Further non-bonding interactions, included Pi–Pi T-shaped interactions with PHE834. In the same way, the binding interactions of **S14** involved two hydrogen bonds between the acyl group and ASP833, VAL700. The pi–sigma, pi–sulfur, and pi–alkyl bonds, along with Van der Waals forces, were formed between **S14** and LYS723, VAL704, CYS721 and ARG819. The binding interactions of the reference drug and compounds **S11** and **S14** are shown in Figure 5.

#### 2.7.2. Molecular Interactions with VEGFR2

**Figure 6:**
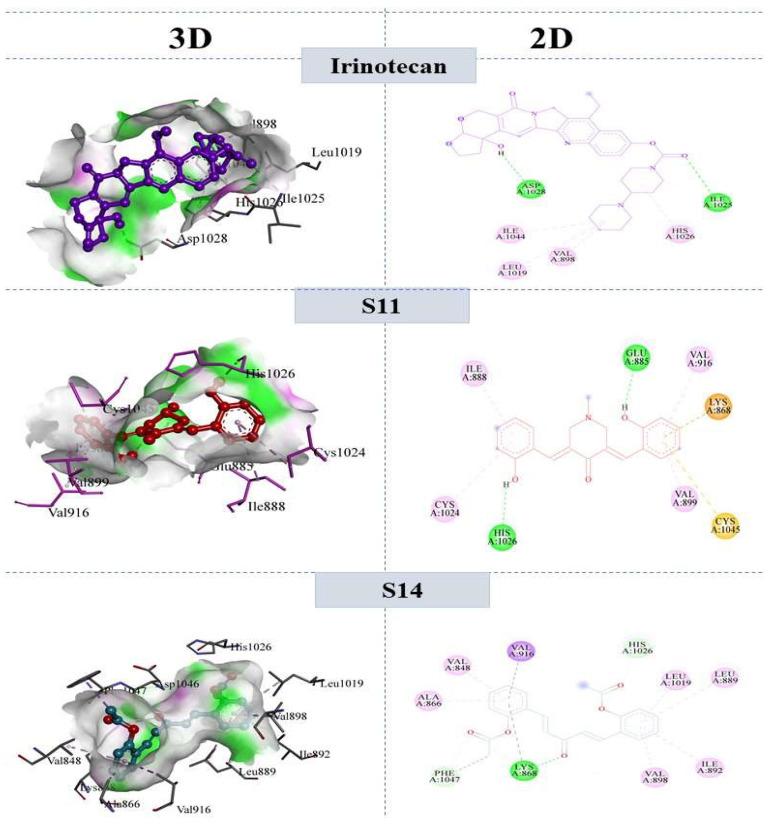
3D and 2D interaction of reference compound Irinotecan, **S11** and **S14** within the active pocket of VEGFR-2 tyrosine kinase.

The docked conformation of standard irinotecan and curcumin derivatives revealed substantial molecular interactions with ERBB and VEGFR2. From the analysis of docking interactions, it was revealed that the two hydroxyl groups of **S11** formed two strong hydrogen bonds with GLU 885 and HIS 1026, and all other interactions were weak pi–cations, pi–sulfur, and pi–sigma bonds. The bonding and non-bonding interactions of the most potent derivative, i.e., **S14**, involved the following amino acids: VAL848; VAL916; ALA866; HIS1026; LEU1019; LEU889; ALA866; PHE1047; LYS868; ILE892. The strongest hydrogen bond among the bonding interactions was established between the oxygen atom of the acyl group and LYS 868, whereas the second hydrogen bond was created between the carbon atom and PHE 1047. In addition to these bonds, various pi–alkyl, pi–sigma, and Van der Waals forces were also present. The binding interactions of the reference drug and compounds **S11** and **S14** are shown in Figure 6.

### 2.8. Molecular Dynamics Simulations

#### 2.8.1. MD Simulation Studies of VEGFR2 and Compound **S14**

The molecular dynamics simulations were performed for evaluation of steadfastness of protein–ligand complex under accelerated conditions. The top ranked conformations against each enzyme i.e., VEGFR2 and ERBB were retrieved and subjected to evaluation of stability patterns. The analytical metrics including RMSD, RMSF, contact map analysis, interaction timeline and radius of gyration were utilized for interpretation of protein–ligand complex integrity and stability.

**Figure 7:**
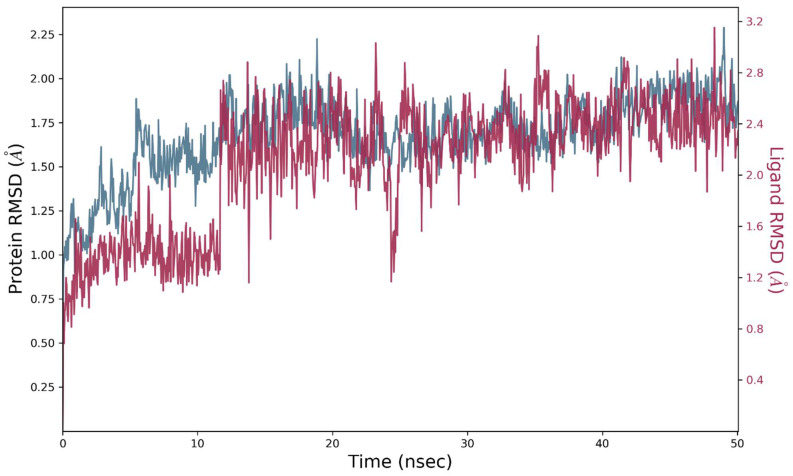
Root mean square deviation (RMSD) of VEGFR2, and VEGFR2-**S14** complex as a function of time. The blue colored trajectory indicates the evolution of RMSD for C alpha atoms, whereas the red trajectory is for the protein–ligand complex.

The MD simulation studies on the VEGFR2-**S14** complex revealed stability patterns for both the apo protein and liganded protein. Concisely, it was notable that the apo protein was extremely stable with an average RMSD of 1.74 angstroms. The RMSD pattern for the apo protein became stable and equilibrated after 10 ns of simulations. In terms of stability pattern of liganded protein, it was observed that liganded protein exhibited modest fluctuations with an average RMSD value of 2.3 angstroms. The slight rearrangement was observed during the initial phase of simulations but after 15 ns, RMSD of liganded protein attained equilibrium and became stable. Moreover, it was notable that ligand remained sufficiently attached to amino acid residues of the active site and produced contacts with shorter bond lengths. The data demonstrate the protein and its associated complex had excellent stability in aqueous media. Figure 7 illustrates the RMSD pattern for the apo and liganded protein.

**Figure 8:**
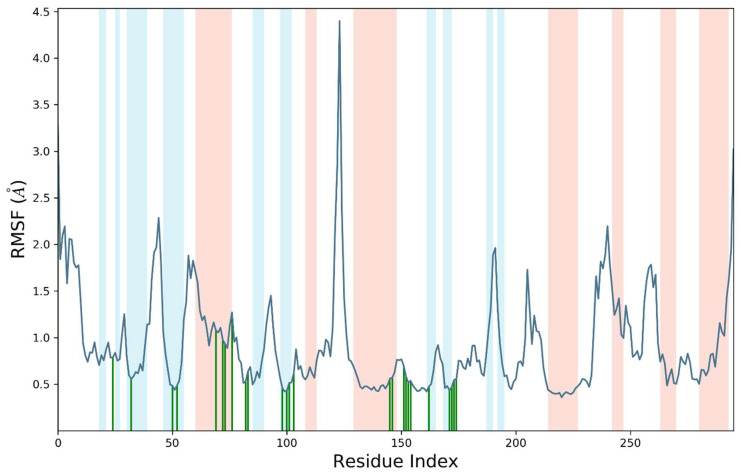
Root mean square fluctuation (RMSF) of Cα atoms of VEGFR2 in complex with **S14**.

The RMSF analysis of liganded protein was conducted for the determination of residue wide fluctuations. The amino acid residues of the VEGFR2 protein exhibited minor variations, especially residues belonging to alpha helix and beta strand were significantly stable. This was expected as both these portions of proteins are rigid and exhibit compactness. The most importantly the amino acid residues of active site (140–170) were in contact with **S14** and exhibited fewer fluctuations. The average RMSF value of the targeted protein was 0.8 angstroms. In addition, amino acid residues belonging to N and C terminals were slightly less compact with higher fluctuations. Figure 8 shows the RMSF value for each residue of the VEGF2 protein.

**Figure 9:**
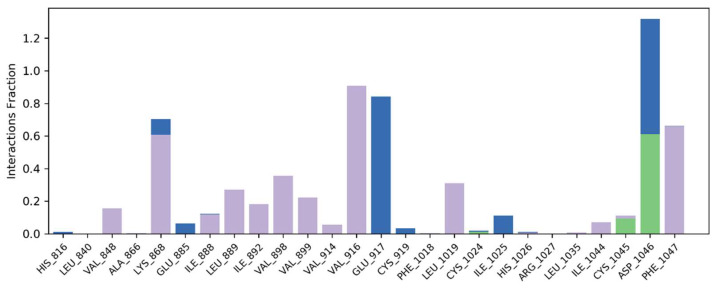

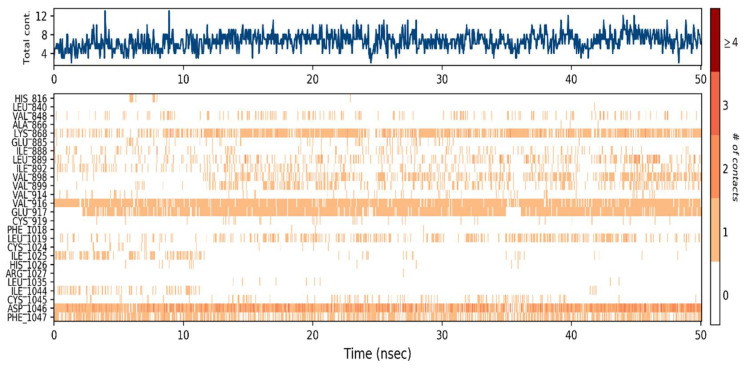
Illustration of contact map histogram and timeline for 50 ns simulations.

Multiple important molecular interactions were produced by **S14** with amino acid residues of the active site. Specifically, amino acid residues VAL848, ILE888, Leu889, ILE892, VAL898, Val899, VAL914, VAL916, LEU1019, ILE1044 and PHE1047 were engaged in hydrophobic interactions. Significant interaction times were observed with LYS868, VAL916 and PHE1047 with interaction times of 60%, 90% and 70%, respectively. Furthermore, two hydrogen bonds exist between ASP1046 and CYS1045, respectively. The interaction fraction of ASP1046 was 60% and 10% for CYS1045. Multiple water bridges were also produced during simulation studies. The contact map histograms and contact map timeline are illustrated in Figure 9.

#### 2.8.2. MD Simulations Analysis of the ERBB–**S14** Complex

**Figure 10:**
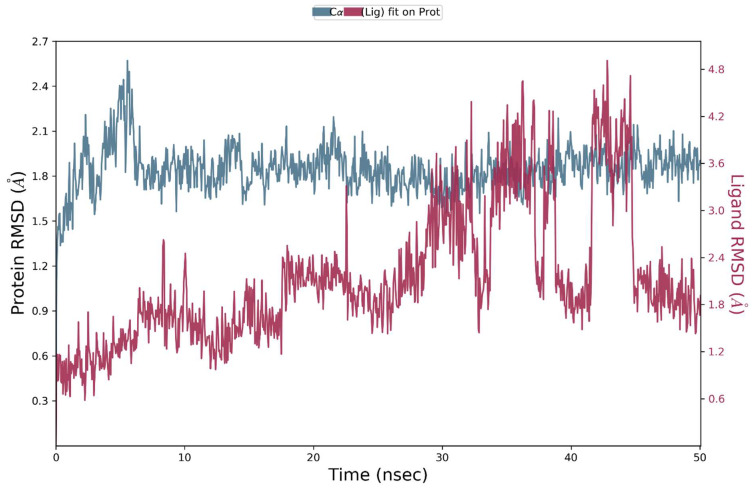
Root mean square deviation (RMSD) of ERBB, and the EGFR–**S14** complex as a function of time. The blue colored trajectory indicates the evolution of RMSD for C alpha atoms, whereas the red trajectory represents the protein–ligand complex.

To study the complex’s molecular dynamics and stability, the ERBB protein’s docked complex with the best pose of **S14** was simulated in an aqueous environment for a 50ns trajectory under periodic boundary conditions. The sole protein and its complex were considered an initial point for MD simulation studies. The RMSD value was calculated for the C alpha atoms and protein–ligand complex (ERBB–**S14**) in order to investigate the stability pattern during simulated trajectory. The RMSD pattern of protein and its complex is presented in Figure 10. The RMSD pattern for c alpha atoms of protein became stable and equilibrated after 5 ns of simulation. Initial fluctuations were observed in C and N terminal residues of ERBB which became stable and equilibrated after 5 ns. The average RMSD value for C alpha atoms was 1.8 angstroms. In contrast, the protein–ligand complex was exhibiting slight rearrangement inside the active pocket of the targeted protein. The protein–ligand complex trajectory was stable and equilibrated after 30 ns of simulation but after that the ligand exhibited rearrangements and produced new contacts with active site residues. These rearrangements lasted for 10 ns, and after that the ligand again became stable and the trajectory became equilibrated toward the end of the simulations. On the basis of these findings, it could be deduced that **S14** could be an effective inhibitor of VEGFR2, whereas there was modest inhibitory potential observed against ERBB. Figure 10 shows the evolution of the RMSD pattern for protein and protein-**S14** complex.

**Figure 11:**
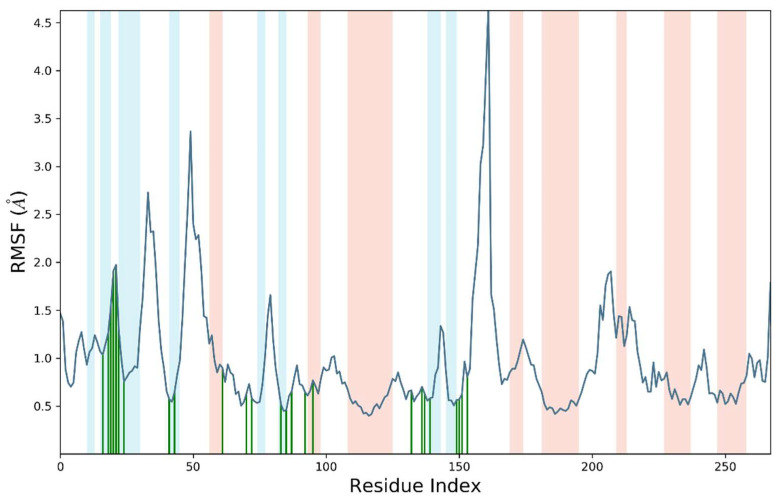
Root mean square fluctuation (RMSF) of Cα atoms of complex ERBB–**S14**.

The perturbation of each amino acid residue was evaluated through RMSF analysis over a 50 ns simulated trajectory. Most of the residues were perturbed below 2 angstroms except amino acid residues ranges from 10–30 and 152 to 160. These residues exhibited fluctuations up to 4 angstroms. In addition, it was notable that important residues were in significant contact with **S14** indicating the compactness of amino acid residues belonging to the active site. The average RMSF value for liganded ERBB protein was 1.1 angstroms. The root mean square fluctuation of liganded protein is illustrated in Figure 11.

**Figure 12:**
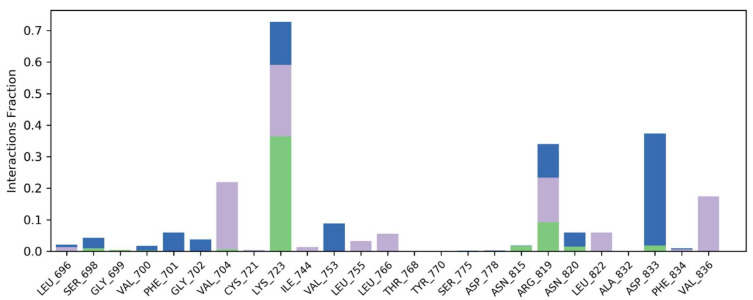

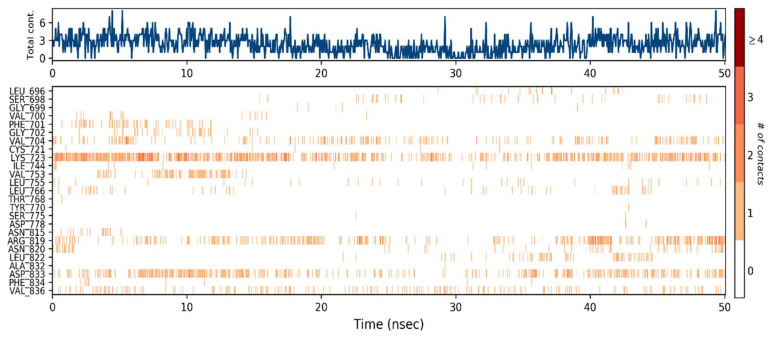
The contact map analysis and timeline of ERBB–**S14** complex.

The contact map analysis and buried surface area was also computed through MD simulations. The important molecular interactions were included hydrophobic and hydrogen bonding interactions. The amino acid residues involved in hydrogen bonding was LYS723 and ARG619 with interaction fraction of 30% and 10% respectively. These residues were buried by **S14** for majority of simulated trajectory. In addition, VAL704, LYS723, ARG619 and VAL836 were engaged in hydrophobic interactions. The interaction fraction of following residues was as follows; 20%, 25%, 10% and 10% respectively. Furthermore, water bridges were also contributing toward stability of protein ligand complex. The contact map histogram and contact timeline is illustrated in Figure 12.

#### 2.8.3. The MMGBSA Free Energy Calculations

The molecular docking provide initial binding energy which provide an estimate of binding affinity between protein and ligand. However, molecular docking is not robust technique in estimating binding free energies. For efficient prediction of binding affinity, MMGBSA analysis were performed which take into account all electrostatic, hydrophilic and hydrophobic interactions and provide cumulative binding free energy [24]. The both complexes were subjected to MMGBSA analysis and provided in Table 11.

The following chemical equation was used to calculate free binding energy calculations [24];

ΔG_bind_ = ΔG_SA_ +ΔG_SOL_ + ΔE_mm_

### 2.9. SeeSAR Analysis

**Figure 13:**
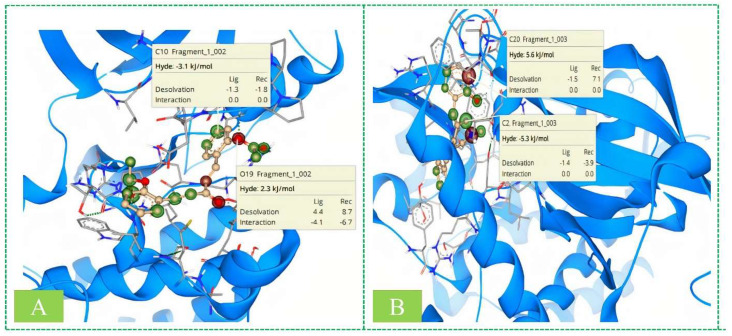
3D (**A**) and 2D (**B**) interaction of derivative **S14** within the active pocket of ERBB and VEGFR2 kinase.

SeeSAR analysis with the most potent derivative was confirmed for ERBB and VEGFR by using SeeSAR by BiosolveIT [25], which visually depicts binding affinity. The HYDE value was calculated, indicating that the green coronas around the atom represent the atoms involved in positively developing the binding affinity; the higher the contribution, the larger the corona size. In the same way, the red-colored coronas around atoms indicated the unfavorable contributions towards binding affinity, and atoms with no significant involvement are not colored. Figure 13 shows the SeeSAR visualization of the most potent inhibitors. Although, as evident from the results, most of the atoms in the molecule contribute favorably to overall binding (indicated by green-colored coronas) in both of the proteins, only two different structural elements are not contributing favorably (indicated by red-colored coronas) because of high desolvation energy.

## 3. Materials and Methods

### 3.1. Generation of Pharmacophore Model

**Figure 14:**
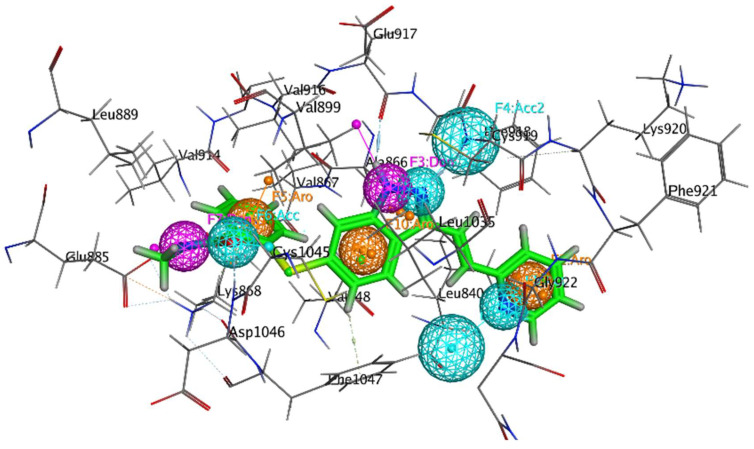
Binding interactions of Axitinib (green sticks) with VEGFR-2. The pharmacophore features are represented as blue, purple, and orange colored spheres.

The current study developed a pharmacophore model for a protein–ligand complex using the pharmacophore query editor wizard of the Molecular Operating Environment (MOE) [26]. The binding interactions of the protein–ligand complex provide initial points for generating chemical features, which were utilized for developing pharmacophore models. MOE makes use of several built-in pharmacophore features, including a hydrogen acceptor (Acc), an anionic atom, a hydrophobic center, an aromatic center (Ar), a cationic atom, and a hydrogen bond donor (Don) [27]. In the current layout, only important chemical features, i.e., hydrogen bond acceptor, hydrogen bond donor, and hydrophobic interactions, were used to develop the pharmacophore model. The PDB ID 4AG8 was used to retrieve the crystal structure of VEGFR-2 in complex with Axitinib (N-methyl-2-[[3-[(E)-2-pyridin-2-ylethenyl]-1H-indazol-6-yl]sulfanyl]benzamide). The crystallographic complex was utilized for the generation of pharmacophore features. Axitinib produced strong interactions with amino acid residues of VEGFR-2. Important amino acid residues and pharmacophore features of Axitinib are shown in Figure 14.

It is crucial to validate the created pharmacophore model by screening the decoy molecules and known inhibitors of the targeted protein. The PubChem database retrieved ten known inhibitors of the targeted proteins and tested them against the created pharmacophore model.

### 3.2. Pharmacophore-Based Virtual Screening

Following the generation of a pharmacophore query model, a total of 14 curcumin derivatives were subjected to screening against the developed model. Only those derivatives that satisfied the pharmacophore features criteria were considered hit molecules. These models are essential for discovering novel molecules and are also crucial for anti-target modeling to avoid any adverse effects. In order to validate the generated pharmacophore model, a test dataset comprised of ten reported inhibitors of VEGFR2 (including sorafenib) and ten decoy molecules was constructed and virtually screened against the constructed pharmacophore model. The validated model was then subjected to pharmacophore based screening of curcumin derivatives. The Pharmacophore-based screening is superior to docking when structural information about the target protein or ligand’s active conformation is present. Finally, the hit molecules obtained via pharmacophore-based virtual screening were processed further for detailed in-silico investigation.

### 3.3. Density Functional Theory (DFTs)

The geometric parameters and structural geometries of curcumin derivatives were evaluated/optimized through density functional theory calculations. The density functional theory (DFTs) calculations were performed using the Guassian09W program [28]. The accurate assumptions and structural convergence were achieved through B3LYP functional correlation, and 3-21G as a basis set [29]. The 3-21G was opted as a basis set which offered multiple functions including s and p functions for accurate prediction of electronic properties of compounds. Moreover, 3-21G is commonly employed for fast and accurate assumptions on electron density of compounds. Using the proposed approach, the comprehensive reactivity profile of each compound was evaluated through various matrices including frontier molecular orbitals (FMO) analysis, global and local reactivity descriptor and electrostatic potential map. The resultant output files were analyzed through Guass View 6 [30].

### 3.4. Filtration for Drug-Likeness and Virtual Screening

The safety profile of a drug candidate is paramount in determining the fate of drug discovery and the drug development process. A drug that needs to be administered in the human body must have sufficient absorption, distribution, metabolism, and excretion properties. The in-silico ADMET is a crucial step in the drug discovery process that determines the safety and toxic profile of a drug candidate. The comprehensive pharmacokinetic and safety profiles of selected derivatives were determined via an in-silico approach. The online web server tool, ADMET Lab 2.0, was utilized to predict various physicochemical properties, i.e., ADMET (absorption, distribution, metabolism, excretion, and toxicity) properties [31].

### 3.5. Molecular Docking Studies

The molecular docking studies were performed on the optimized structures of curcumin derivatives obtained from DFT studies. Molecular Operating Environment (MOE) and AutoDock 4.0 were used to perform molecular docking experiments [26,32,33]. The two docking programs were employed in order to enhance the accuracy of the docking protocol. Both software packages were evaluated for their dependability and ability to regenerate docked conformations, and the program that performed best was chosen for additional molecular docking research. The re-docking of all the compounds was carried out using MOE because of its high reliability. For molecular docking studies, two steps are mandatory, i.e., ligand and protein preparations. Each ligand underwent a superficial energy minimization process to begin the docking process using ChemDraw 3D software. Following that, the atomic charges and the potential energy were added. Additionally, various properties of the ligands were measured using the MMFF94x force field [30], and the ligand library was then saved in the required format (MDB). The targeted protein structures were downloaded from the RCSB protein data bank (www.rcsb.com accessed on 1 September 2022) with PDB IDs: 3LMG (ERBB tyrosine kinase), 3HNG (VEGFR1 kinase), 4BSJ (VEGFR3 kinase) and 4AG8 (VEGFR2 kinase) [34]. The first step in protein preparation is adding polar hydrogen atoms to the active sites, followed by potential energy fixation. The protein active pocket is then identified using MOE’s built-in site finder, followed by chain type selection. Finally, the two critical components (ligand and protein) are ready to commence the docking process. For each ligand, 30 poses were generated to identify the most stable configuration of the complex. The current study has utilized the London dG scoring function to analyze the interaction efficiency and adjusted it twice using triangular matcher methods. At the end of the process, important docking interaction data, i.e., receptor interactions, associated amino-acid residues, binding energy, and type of interactions, were recorded [25]. The Biovia Discovery Studio Visualizer (2020) and the MOE’s inbuilt visualization tool were used to analyze all docking results. The docking results were validated based on the RMSD value, i.e., any pose with low binding energy and an RMSD value of less than 2.0 was considered the best pose.

### 3.6. Molecular Dynamics Simulation Studies

The molecular dynamic study of the best-docked conformation was performed using Desmond software on a CUDA-accelerated GPU system having a 16 core processor and 64 GB Ram memory. A maestro graphical user interface was used to visualize the results of MD simulations [25]. MD simulations were done to determine how binding works and how stable the protein–ligand complex is under fast conditions. Using the OPLS3 forcefield, the best-docked protein–ligand complexes were chosen, and topology files were made for both the protein and the ligand [35]. By adding NaCl charges at a standard concentration of 0.15 M, the system was brought back to a neutral state. The energy gradient was made as steep as possible to eliminate any close contact between atoms. The system was brought into balance in the NVT ensemble for 500,000 steps, then in the NPT ensemble for another 500,000 steps. After that, the simulation was run for 50 ns with periodic boundaries [36]. The PME method [37] was used to figure out the binding energy, Van der Waals forces, and electrostatic interactions. The SeeSAR analysis was also presented in the current study to evaluate the binding affinities of protein–ligand complexes [38].

### 3.7. Compound Similarity Index

The present work also focused on determining the similarity index between FDA-approved Axitinib and top hits obtained via pharmacophore-based virtual screening. Similarity index and structural activity relationship drug design approaches are based on the assumption that molecules with high similarity index compounds have similar properties and similar biological activities. In this context, the current study has investigated the similarity index between Axitinib with known biological activity against a set of curcumin derivatives. The similarity index was quantified using two different similarity coefficients, i.e., the Tanimoto and Dice index [39].

## 4. Conclusions

Comprehensive in-silico investigations on previously reported anti-cancer derivatives were performed in the current study to discover potent hits of ERBB and VEGFR-2. Initially, pharmacophore-based virtual screening was conducted. Afterward, the optimization and frequency calculations of selected compounds were carried out using DFT studies, and the optimized structures were further subjected to molecular docking studies. The molecular dynamic simulations were conducted further to support the findings of molecular docking. The compounds **S11** and **S14** were identified as potent ERBB and VEGFR2 inhibitors whereas compound **S8** was predicted as a potential inhibitor of VEGFR1 and VEGFR3. The ADMET properties, MD simulations, and SeeSAR analysis confirmed the study’s findings, demonstrating that the selected compounds can be used for further experimental validation. Based on these findings, it is concluded that curcumin derivatives have a strong inhibitory potential against VEGFR1, VEGFR3, VEGFR2 and the ERBB protein, and that they can be used to treat cancer and its associated malignancies. As the current study is based on pure computational investigations, further in-vitro and in-vivo studies are recommended to develop safe and effective inhibitors of cancer proteins.

## Figures and Tables

**Table 1:** Binding energies of all derivatives against VEGFR1, VEGFR2, VEGFR3 and ERBB tyrosine kinase.

Compound	IUPAC Formula	ERBB Binding Energies (kJ/mol)	VEGFR2 Binding Energies (kJ/mol)	VEGFR3 Binding Energies (kJ/mol)	VEGFR1 Binding Energies (kJ/mol)
**S1**	1E,6E)-1,7-bis(4-hydroxy-3-methoxyphenyl)hepta-1,6-diene-3,5-dione	−27.65	−33.4	−22.8	−34.16
**S2**	(2E,6E)-2,6-bis(4-hydroxy-3-methoxybenzylidene)cyclohexanone	−26.32	−29.56	−24.76	−35.28
**S3**	(1E,4E)-1,5-bis(3,4-dimethoxyphenyl)penta-1,4-dien-3-one	25.74	−32.67	−17.6	−35.1
**S4**	(1E,4E)-1,5-bis(2-methoxyphenyl)penta-1,4-dien-3-one	−25.51	−28.20	−22.92	−32.44
**S5**	(1E,4E)-1,5-bis(3-hydroxyphenyl)penta-1,4-dien-3-one	−23.79	−26.9	−22.68	−34.96
**S6**	(1E,4E)-1,5-bis(2-fluorophenyl)penta-1,4-dien-3-one	−24.64	−32.4	−19.08	−31.2
**S7**	(1E,4E)-1,5-bis(2-hydroxyphenyl)penta-1,4-dien-3-one	−26.54	−37.5	−21.72	−30.5
**S8**	(1E,4E)-1,5-bis(2-hydroxyphenyl)penta-1,4-dien-3-one	−25.87	−32.7	−29.88	−41.48
**S9**	(3E,5E)-3,5-bis(2-hydroxybenzylidene)dihydro-2H-pyran-4(3H)-one	−27.37	−36.3	−29.24	−40.84
**S10**	(3E,5E)-3,5-bis(2-fluorobenzylidene)dihydro-2H-pyran-4(3H)-one	−25.87	−37.2	−22.36	−15.2
**S11**	(3E,5E)-3,5-bis(2-hydroxybenzylidene)-1-methylpiperidin-4-one	−37.92	−43.2	−28.04	−36.2
**S12**	(3E,5E)-3,5-bis(2-fluorobenzylidene)-1-(1-hydroxy-2-oxopropyl)piperidin-4-one	−31.68	−38.78	−26.1	−32.68
**S13**	(1E,4E)-1,5-bis(4-hydroxyphenyl)penta-1,4-dien-3-one	−29.41	−33.95	−20.52	−32.8
**S14**	((1E,4E)-3-oxopenta-1,4-diene-1,5-diyl)bis(2,1-phenylene) diacetate	−38.5	−46.5	−22.24	−35.2
Irinotecan (reference)	4,11-diethyl-4-hydroxy-3,14-dioxo-3,4,12,14-tetrahydro-1H-pyrano [3’,4’:6,7]indolizino [1,2-b]quinolin-9-yl [1,4’-bipiperidine]-1’-carboxylate	−28.4	−40.24	−38.32	−41.21

**Table 2:** Similarity index values.

Compound	Tanimoto MACCS	Tanimoto Morgan	Dice MACCS	Dice Morgan
**S11**	0.300	0.271	0.461	0.426
**S14**	0.215	0.251	0.354	0.401
**Axitinib**	1.000	1.000	1.000	1.000

**Table 3:** Optimization, Energy Polarizability and Dipole Moment of Compounds in the Gaseous Phase.

Compounds	Optimization Energy (Hatree)	Polarizability (α.u)	Dipole Moment (Debye)
**S1**	−1256.586	257.490	6.373
**S2**	−1220.906	289.801	6.587
**S3**	−1182.989	285.627	4.918
**S4**	−955.207	233.085	3.816
**S5**	−877.013	204.518	4.692
**S6**	−924.814	193.712	2.963
**S7**	−877.024	215.917	1.545
**S8**	−993.128	239.791	1.551
**S9**	−1028.810	233.923	0.569
**S10**	−1076.612	222.898	4.450
**S11**	−1048.146	241.779	1.991
**S12**	−1322.551	259.230	4.842
**S13**	−877.021	228.199	5.811
**S14**	−1180.671	264.307	1.225

**Table 4:** Global reactive descriptors for curcumin analogs.

Compound	Hardness (η)	Softness (S)	Electronegativity (X)	Chemical Potential (μ)	Electrophilicity Index (ω)
**S1**	0.071	0.285	1.138	−1.138	9.107
**S2**	0.069	0.276	1.105	−1.105	8.838
**S3**	0.064	0.256	1.023	−1.023	8.186
**S4**	0.072	0.289	1.156	−1.156	9.248
**S5**	0.073	0.290	1.160	−1.160	9.282
**S6**	0.076	0.303	1.212	−1.212	9.696
**S7**	0.071	7.04	0.142	−0.142	0.142
**S8**	0.070	7.14	0.130	−0.130	0.121
**S9**	0.070	7.14	0.140	−0.140	0.140
**S10**	0.075	6.67	0.155	−0.155	0.160
**S11**	0.068	7.35	0.132	−0.132	0.128
**S12**	0.067	7.46	0.147	−0.147	0.161
**S13**	0.069	7.25	0.142	−0.142	0.146
**S14**	0.073	6.90	0.155	−0.155	0.165

**Table 5:** Various global descriptors and their calculated values for selected compounds.

Codes	E_HOMO_ (eV)	E_LUMO_ (eV)	∆E_gap_ (eV)	Potential Ionization I(eV)	Affinity A(eV)	Electron Donating Power (ω−)	Electron Accepting Power (ω+)	Electrophilicity (Δω±)
**S1**	−0.21	−0.06	0.142	0.21	0.06	3.11	8.41	11.53
**S2**	−0.20	−0.06	0.138	0.20	0.06	3.02	8.17	11.19
**S3**	−0.19	−0.06	0.128	0.19	0.06	2.82	7.68	10.51
**S4**	−0.22	−0.07	0.145	0.22	0.07	3.19	8.66	11.85
**S5**	−0.22	−0.07	0.145	0.22	0.07	3.21	8.74	11.95
**S6**	−0.24	−0.08	0.152	0.24	0.08	3.38	9.28	12.67
**S7**	−0.213	−0.071	0.142	0.213	0.071	0.222	0.080	0.302
**S8**	−0.20	−0.06	0.140	0.20	0.06	0.194	0.064	0.259
**S9**	−0.21	−0.07	0.140	0.21	0.07	0.219	0.079	0.298
**S10**	−0.23	−0.08	0.150	0.23	0.08	0.247	0.092	0.339

**Table 6:** Various physicochemical properties of selected compounds.

Physicochemical Properties								
	Molecular Weight	Density	nHA	nHD	TPSA	LogS	LogP	LogD
**S1**	368.13	0.966	6	2	93.06	−3.921	2.742	2.82
**S2**	366.15	0.954	5	2	75.99	−4.475	3.441	3.437
**S3**	354.15	0.945	5	0	53.99	−5.442	3.118	3.271
**S4**	294.13	0.911	3	0	35.53	−5.88	3.808	3.775
**S5**	266.09	0.924	3	2	57.53	−4.003	3.08	3.248
**S6**	270.09	0.955	1	0	17.07	−6.046	3.937	3.963
**S7**	266.09	0.924	3	2	57.53	−4.003	3.08	3.248
**S8**	306.13	0.924	3	2	57.53	−4.2	4.425	3.717
**S9**	308.1	0.954	4	2	66.76	−3.678	3.233	3.156
**S10**	312.1	0.983	2	0	26.3	−5.847	3.904	3.815
**S11**	321.14	0.938	4	2	60.77	−3.052	3.521	3.108
**S12**	383.13	0.991	4	1	57.61	−4.067	3.052	3.437
**S13**	266.09	0.924	3	2	57.53	−3.277	3.201	3.397
**S14**	350.12	0.947	5	0	69.67	−5.518	3.123	2.829

**Table 7:** Absorption and distribution properties of selected compounds.

Absorption and Distribution Properties								
	Volume of Distribution (vd)	Human Intestinal Absorption (hia)	Caco-2 Permeability	Blood Brain Barrier (bbb) and Blood-Placenta Barrier (bpb)	Plasma Protein Binding (ppb)	pgp-Inhibitor	p-Glycoprotein Substrate (pgp-Substrate)	MDCK Permeability
**S1**	2.52	0.008	−4.668	0.218	99.08%	0.087	0.001	1.3 × 10^−5^
**S2**	2.442	0.006	−4.584	0.176	99.53%	0.008	0.001	1.3 × 10^−5^
**S3**	2.398	0.006	−4.604	0.185	99.73%	0.057	0.003	1.2 × 10^−5^
**S4**	2.505	0.006	−4.589	0.165	99.52%	0.069	0.002	1.2 × 10^−5^
**S5**	2.243	0.006	−4.581	0.187	96.94%	0.004	0.004	1.3 × 10^−5^
**S6**	2.155	0.005	−4.513	0.172	98.12%	0.045	0.004	1.2 × 10^−5^
**S7**	2.179	0.005	−4.508	0.161	100%	0.056	0.005	1.2 × 10^−5^
**S8**	2.817	0.005	−4.548	0.159	99.99%	0.007	0.002	1.3 × 10^−5^
**S9**	2.418	0.006	−4.611	0.16	100%	0.015	0.008	1.2 × 10^−5^
**S10**	1.701	0.005	−4.557	0.13	100%	0.33	0.015	1.1 × 10^−5^
**S11**	2.964	0.005	−4.539	0.167	99.86%	0.057	0.001	1.3 × 10^−5^
**S12**	2.846	0.006	−4.542	0.166	99.94%	0.341	0.002	1.2 × 10^−5^
**S13**	2.629	0.005	−4.513	0.077	99.89%	0.717	0.001	1.3 × 10^−5^
**S14**	2.854	0.006	−4.539	0.545	100%	0.627	0.001	1.2 × 10^−5^

**Table 8:** Metabolism and excretion values of selected compounds.

Metabolism							Excretion
Codes	CYP1A2 Inhibitor	CYP2C19 Inhibitor	CYP2C9 Inhibitor	CYP2D6 Inhibitor	CYP3A4 Inhibitor	CL (mL/min)	T1/2 (Hours)
**S1**	0.593	0.287	0.661	0.037	0.674	13.839	0.948
**S2**	0.851	0.823	0.689	0.421	0.6	7.792	0.89
**S3**	0.511	0.478	0.148	0.009	0.474	10.232	0.785
**S4**	0.978	0.904	0.626	0.256	0.883	7.828	0.431
**S5**	0.987	0.421	0.447	0.64	0.939	12.32	0.912
**S6**	0.98	0.742	0.512	0.366	0.254	5.519	0.106
**S7**	0.987	0.421	0.447	0.64	0.939	12.32	0.912
**S8**	0.963	0.958	0.894	0.947	0.477	5.072	0.657
**S9**	0.945	0.884	0.867	0.554	0.282	6.894	0.835
**S10**	0.919	0.946	0.837	0.03	0.055	6.738	0.056
**S11**	0.703	0.741	0.433	0.944	0.062	17.699	0.799
**S12**	0.435	0.868	0.759	0.159	0.054	12.063	0.169
**S13**	0.829	0.636	0.432	0.195	0.558	13.675	0.937
**S14**	0.976	0.94	0.857	0.498	0.206	1.413	0.813

**Table 9:** Medicinal properties and toxicity profile of selected compounds.

Medicinal Properties			Toxicity				
	Synthetic Accessibility Score	Lipinski Rule	AMES Toxicity	Carcinogenicity	Eye Corrosion	Eye Irritation	Respiratory Toxicity
**S1**	2.426	Accepted	0.234	0.706	0.007	0.792	0.951
**S2**	2.408	Accepted	0.103	0.78	0.004	0.303	0.905
**S3**	2.095	Accepted	0.19	0.828	0.02	0.404	0.516
**S4**	2.048	Accepted	0.187	0.636	0.581	0.988	0.688
**S5**	2.269	Accepted	0.083	0.517	0.767	0.99	0.917
**S6**	2.143	Accepted	0.049	0.708	0.651	0.98	0.622
**S7**	2.269	Accepted	0.083	0.517	0.767	0.99	0.917
**S8**	2.394	Accepted	0.638	0.347	0.011	0.952	0.514
**S9**	2.605	Accepted	0.913	0.272	0.013	0.936	0.633
**S10**	2.512	Accepted	0.911	0.887	0.004	0.216	0.666
**S11**	2.555	Accepted	0.724	0.239	0.004	0.018	0.861
**S12**	3.264	Accepted	0.55	0.608	0.003	0.008	0.713
**S13**	2.148	Accepted	0.131	0.463	0.051	0.978	0.947
**S14**	2.345	Accepted	0.632	0.44	0.857	0.988	0.801

**Table 10:** Various toxicological parameters of selected compounds.

TOX21 Pathway				
Compound	NR-AR	NR-AR-LBD	NR-ER	Antioxidant Response Element
**S1**	0.807	0.787	0.6	0.891
**S2**	0.772	0.121	0.926	0.977
**S3**	0.381	0.906	0.892	0.91
**S4**	0.429	0.954	0.964	0.933
**S5**	0.484	0.973	0.975	0.967
**S6**	0.005	0.935	0.625	0.84
**S7**	0.484	0.973	0.975	0.967
**S8**	0.515	0.439	0.932	0.982
**S9**	0.141	0.636	0.908	0.974
**S10**	0.001	0.925	0.447	0.956
**S11**	0.073	0.049	0.422	0.969
**S12**	0.002	0.685	0.113	0.953
**S13**	0.788	0.9	0.981	0.971
**S14**	0.103	0.963	0.751	0.921

**Table 11:** MMGBSA free binding energies calculations.

Complex	*ΔG_bind_ (kcal/mol)*	*ΔE H-bond (kcal/mol)*	*ΔE vdW (kcal/mol)*	*ΔE coulomb (kcal/mol)*
**VEGFR2-S14**	−65.4	−2.1	−44.21	−10.76
**ERBB–S14**	−45.32	−1.4	−28.54	−8.22

## Data Availability

Not applicable.

## Supplementary Materials

The following supporting information can be downloaded at: https://www.mdpi.com/article/10.3390/molecules28104044/s1, Figure S1–S5: 3D and 2D interaction of S1–S14 within the active pocket of VEGFR-1 tyrosine kinase; Figure S6–S10: 3D and 2D interaction of S1–S14 within the active pocket of VEGFR-3 tyrosine kinase; Table S1: Predicted inhibitory constant (ki) [40,41].
