# Supplementary material for: Pharmacophore-Based Virtual Screening and In-Silico Explorations of Biomolecules (Curcumin Derivatives) of Curcuma longa as Potential Lead Inhibitors of ERBB and VEGFR-2 for the Treatment of Colorectal Cancer
Source: Molecules. 2023 May 12;28(10):4044. doi: 10.3390/molecules28104044 (PMC10221949; doi:10.3390/molecules28104044)
Supplement: Supplementary file 1 [file molecules-28-04044-s001.zip › molecules-2169842-supplementary.pdf]

Article

Pharmacophore-based virtual screening and *in-silico* explorations of biomolecules (curcumin derivatives) of curcuma longa as the potential lead inhibitors of ERBB and VEGFR-2 for the treatment of colorectal cancer

Syeda Abida Ejaz [1*], Mubashir Aziz [1], Ammara Fayyaz[1], Mohamed Fawzy Ramadan [2], Muhammad Sajjad Bilal [1]

[1] Department of Pharmaceutical Chemistry, Faculty of Pharmacy, The Islamia University of Bahawalpur, 63100, Pakistan
[2] Department of Clinical Nutrition, Faculty of Applied Medical Sciences, Umm Al-Qura University, Makkah, Kingdom of Saudi Arabia
* **Corresponding Authors**: Syeda Abida Ejaz; abida.ejaz@iub.edu.pk ; abidaejaz2010@gmail.com.Tel: +92-062-9250245 Fax: +92-062-9250245

Supplementary Data

2. Results and Discussion

2.7. Molecular Docking Discussion

The curcumin derivatives were docked against VEGFR1 and VEGFR3. All compounds demonstrated inhibitory potential but compound S8 was most selective and demonstrated highest inhibitory potential against VEGFR1 and VEGFR3. The proteins used for the Molecular Docking of VEGFR1 and VEGFR3 are 3HNG [1] and 4BSJ [2] respectively, taken from Protein Data Bank. The putative 2D and 3D interactions of all derivatives against VEGFR1 and VEGFR3 are shown in figure S1-S10.

2.7.3. Binding interactions of VEGFR-1

Among these derivatives, S8 was found to be the most potent compound for VEGFR1. The amino acid CYS192 formed Conventional Hydrogen bond with cyclohexanone ring. Moreover, the pi-sigma bond formation occurred at the benzene ring with LEU833. Among the hydrophobic (pi-alkyl) interactions included amino acids: VAL841, VAL892, CYS1039 and LYS861 (figure S3).

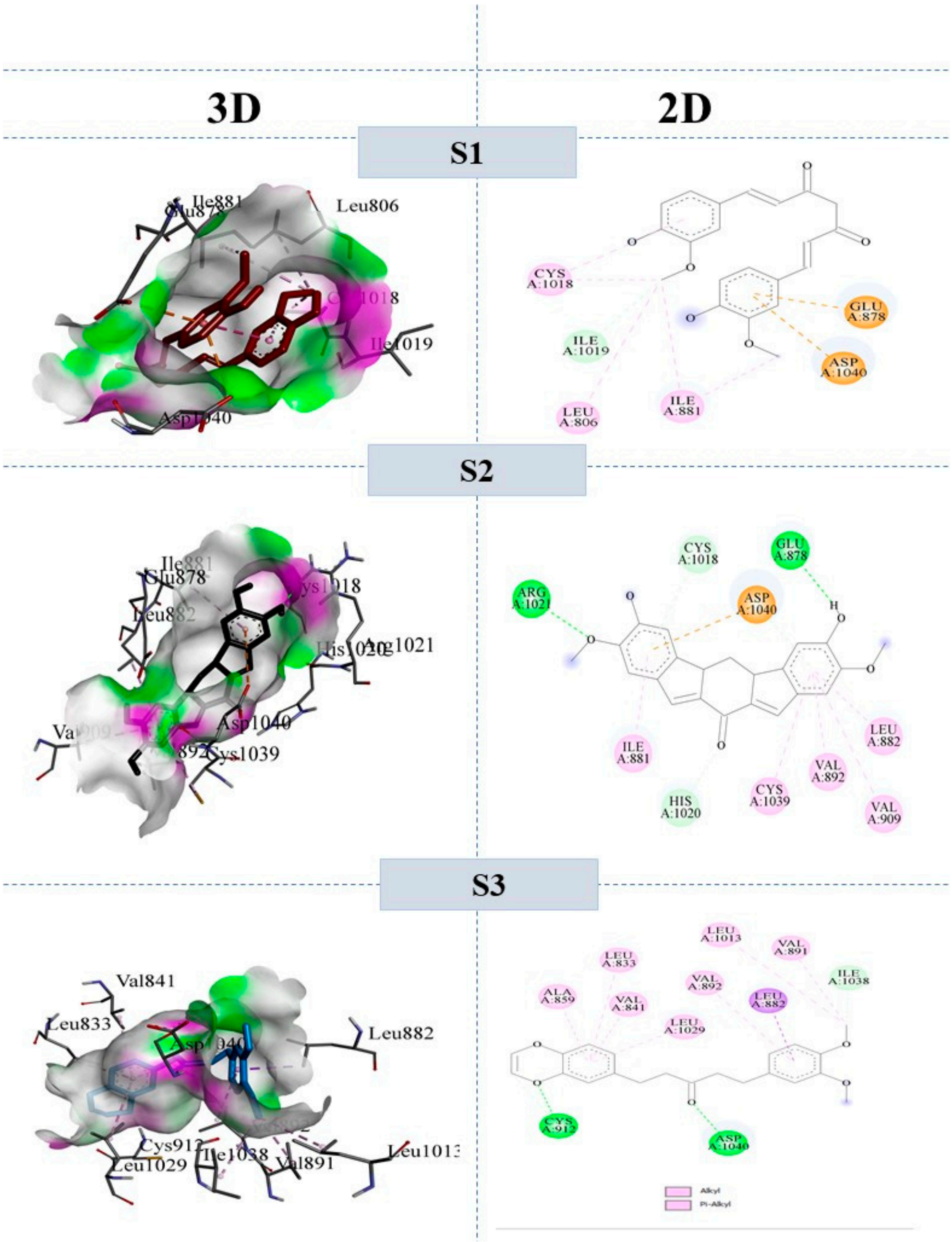

Figure S1. 3D and 2D interaction of S1, S2 and S3 within the active pocket of VEGFR-1 tyrosine kinase.

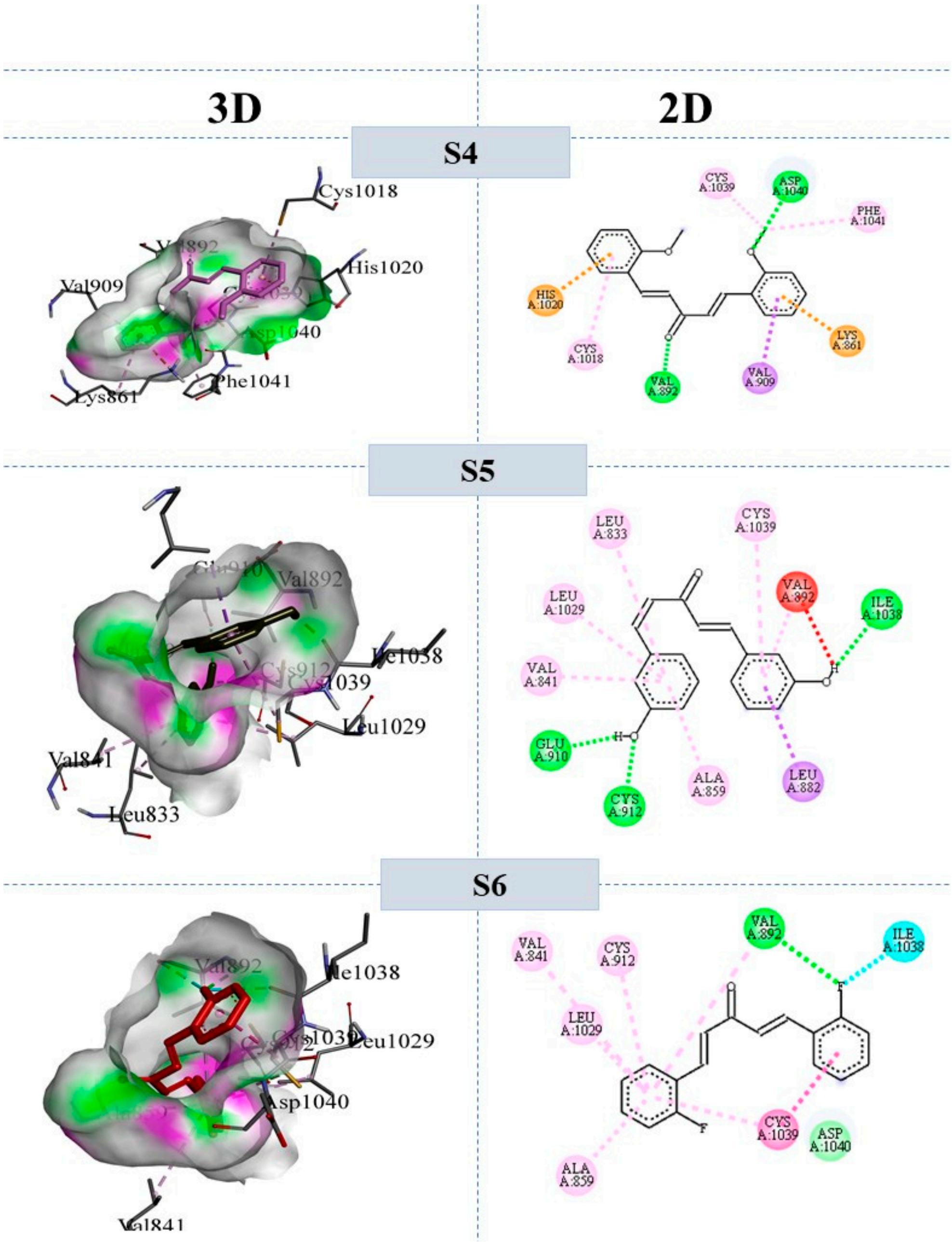

Figure S2. 3D and 2D interaction of S4, S5 and S6 within the active pocket of VEGFR-1 tyrosine kinase.

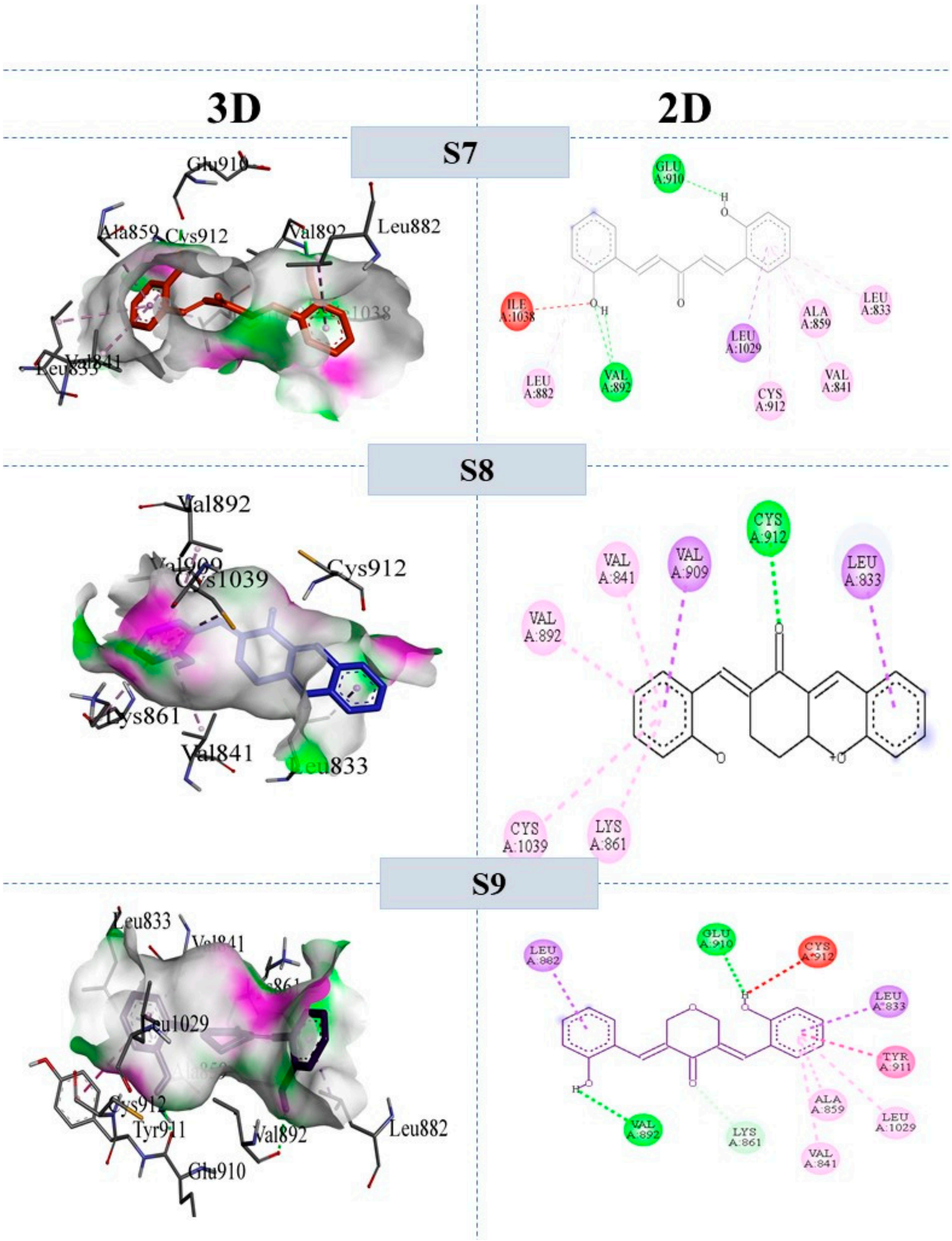

Figure S3. 3D and 2D interaction of S7, S8 and S9 within the active pocket of VEGFR-1 tyrosine kinase.

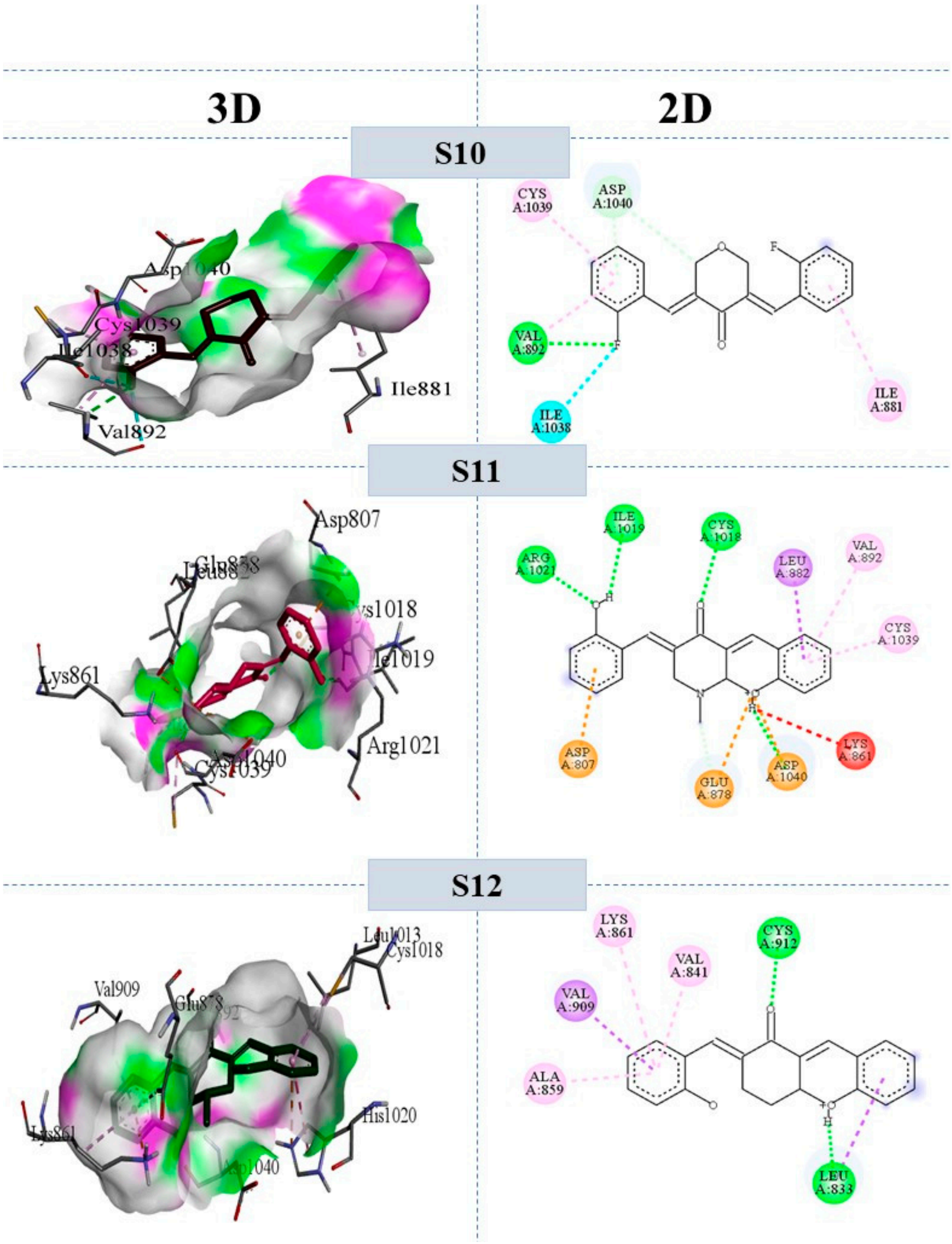

Figure S4. 3D and 2D interaction of S10, S11 and S12 within the active pocket of VEGFR-1 tyrosine kinase.

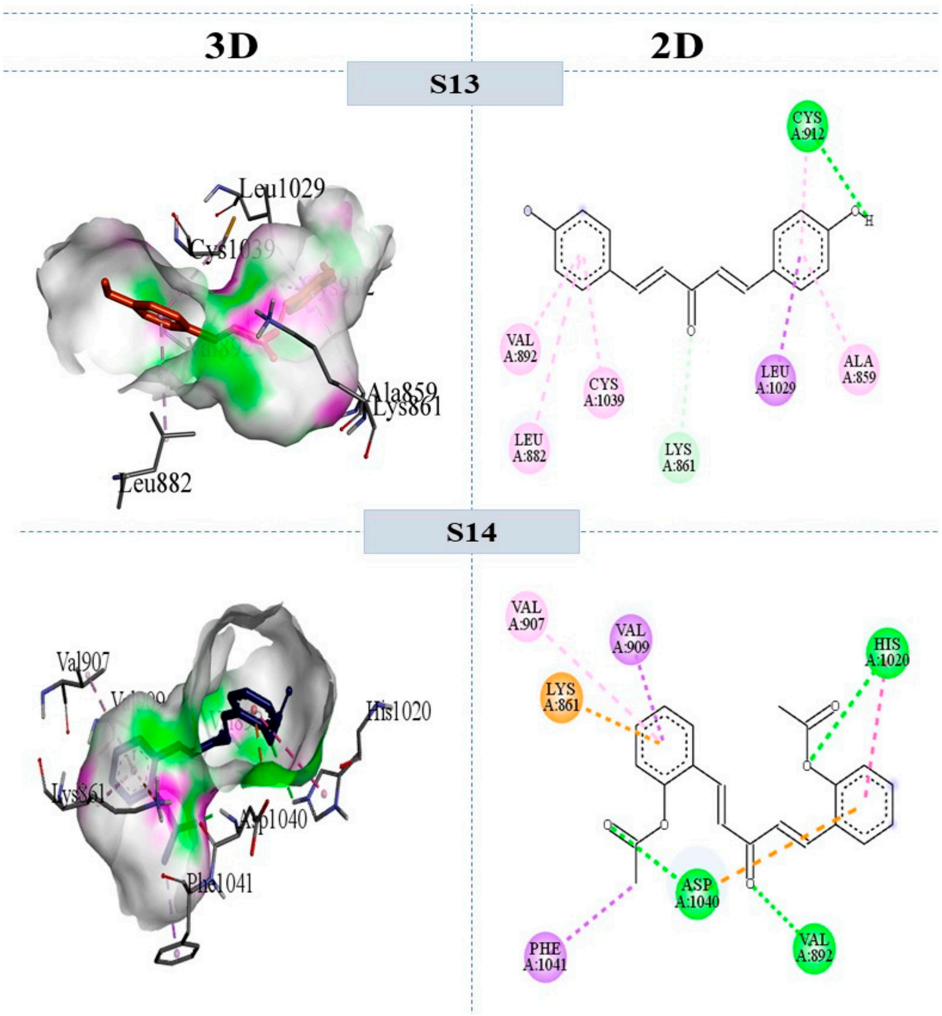

Figure S5. 3D and 2D interaction of S13 and S14 within the active pocket of VEGFR-1 tyrosine kinase.

3.7.4. Binding interactions of VEGFR3

Among these derivatives, S8 was found to be the most potent compound for VEGFR3. The amino acid CYS912, TYR911 formed Conventional Hydrogen bond with the OH groups attached at the Cyclohexane rings. However, there was a major Donor-donor clash at the cyclohexanone ring. Among the hydrophobic (pi-alkyl) interactions included amino acids: ALA859, LEU1029 (Figure S8).

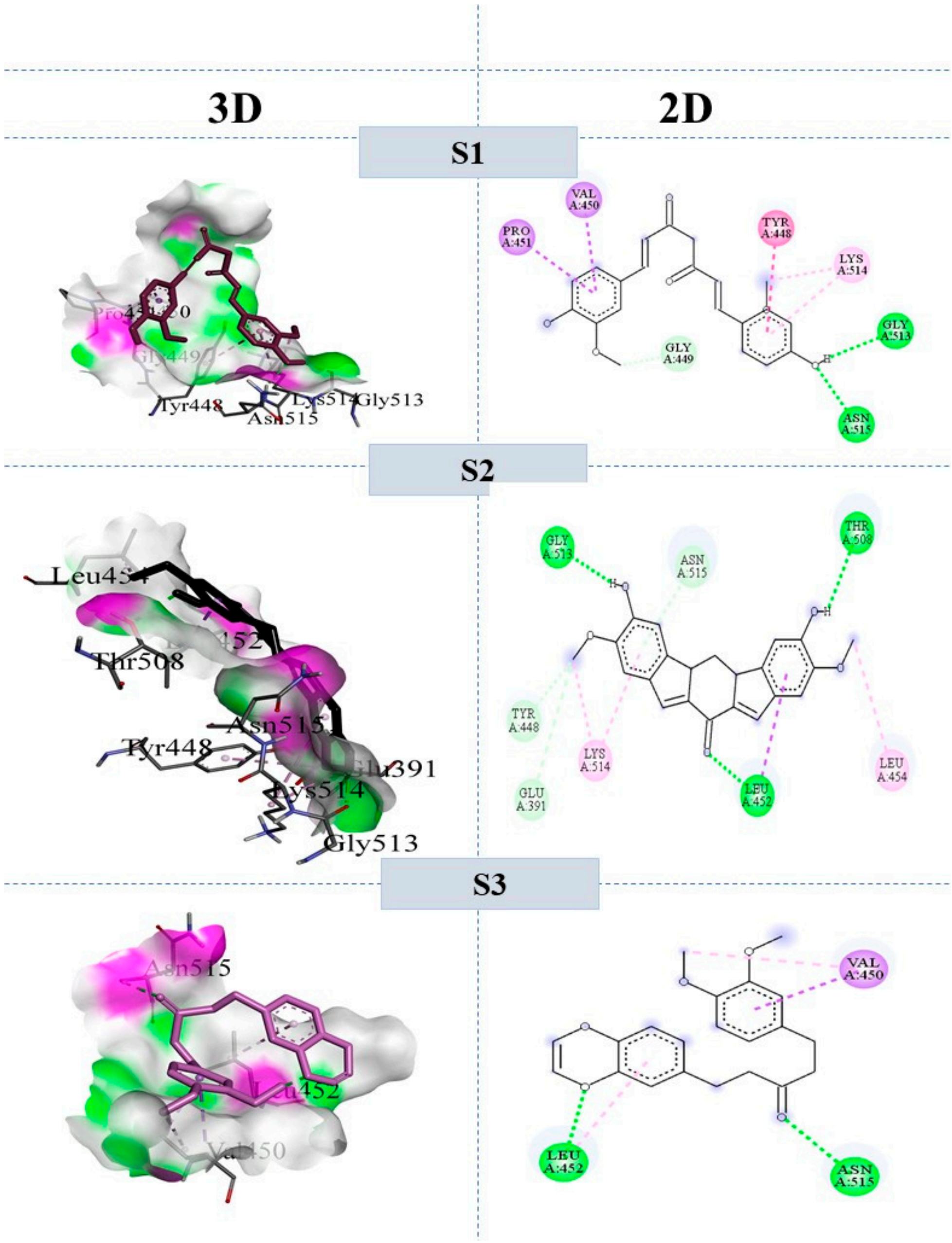

Figure S6. 3D and 2D interaction of S1, S2 and S3 within the active pocket of VEGFR3 tyrosine kinase.

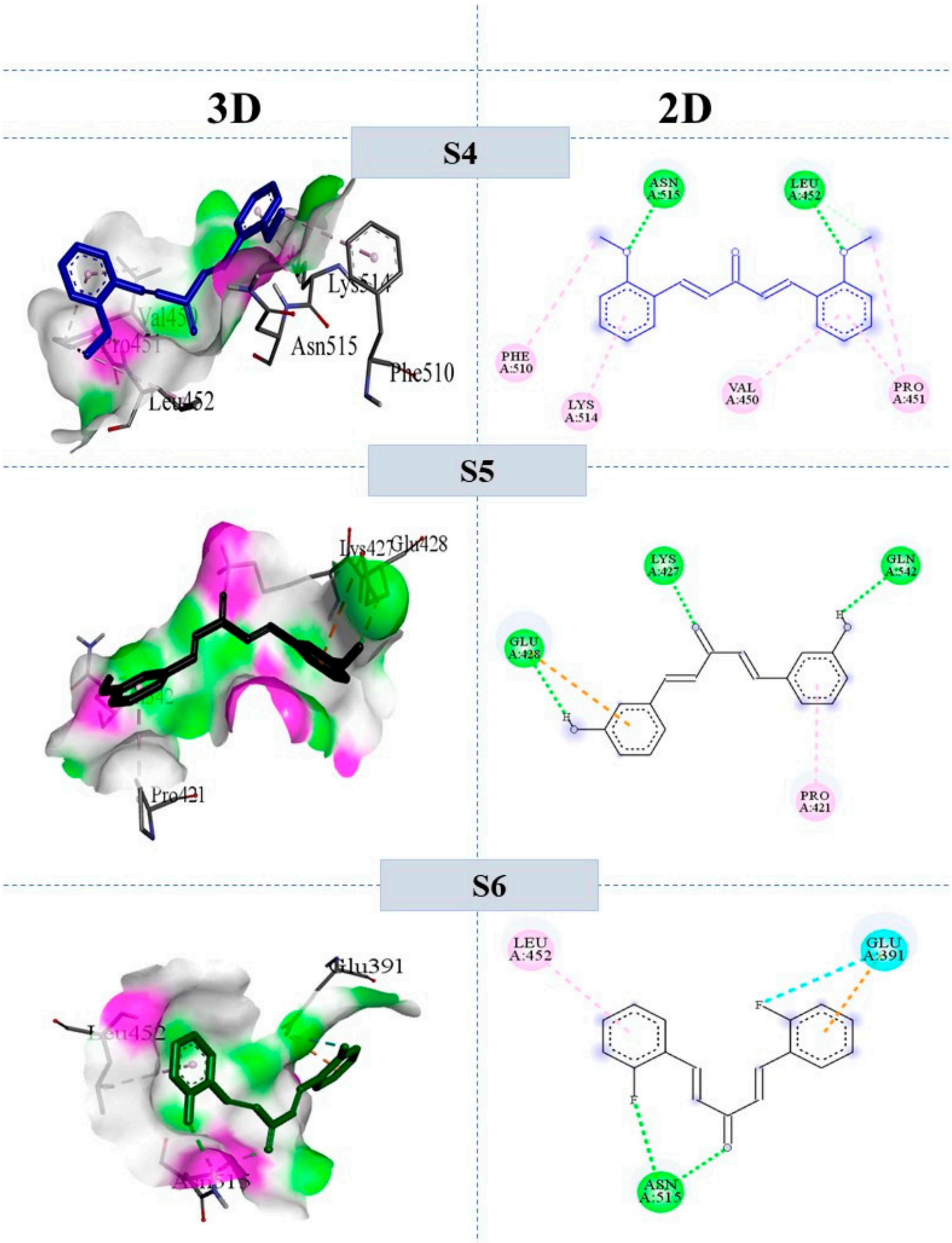

Figure S7. 3D and 2D interaction of S4, S5 and S6 within the active pocket of VEGFR-3 tyrosine kinase.

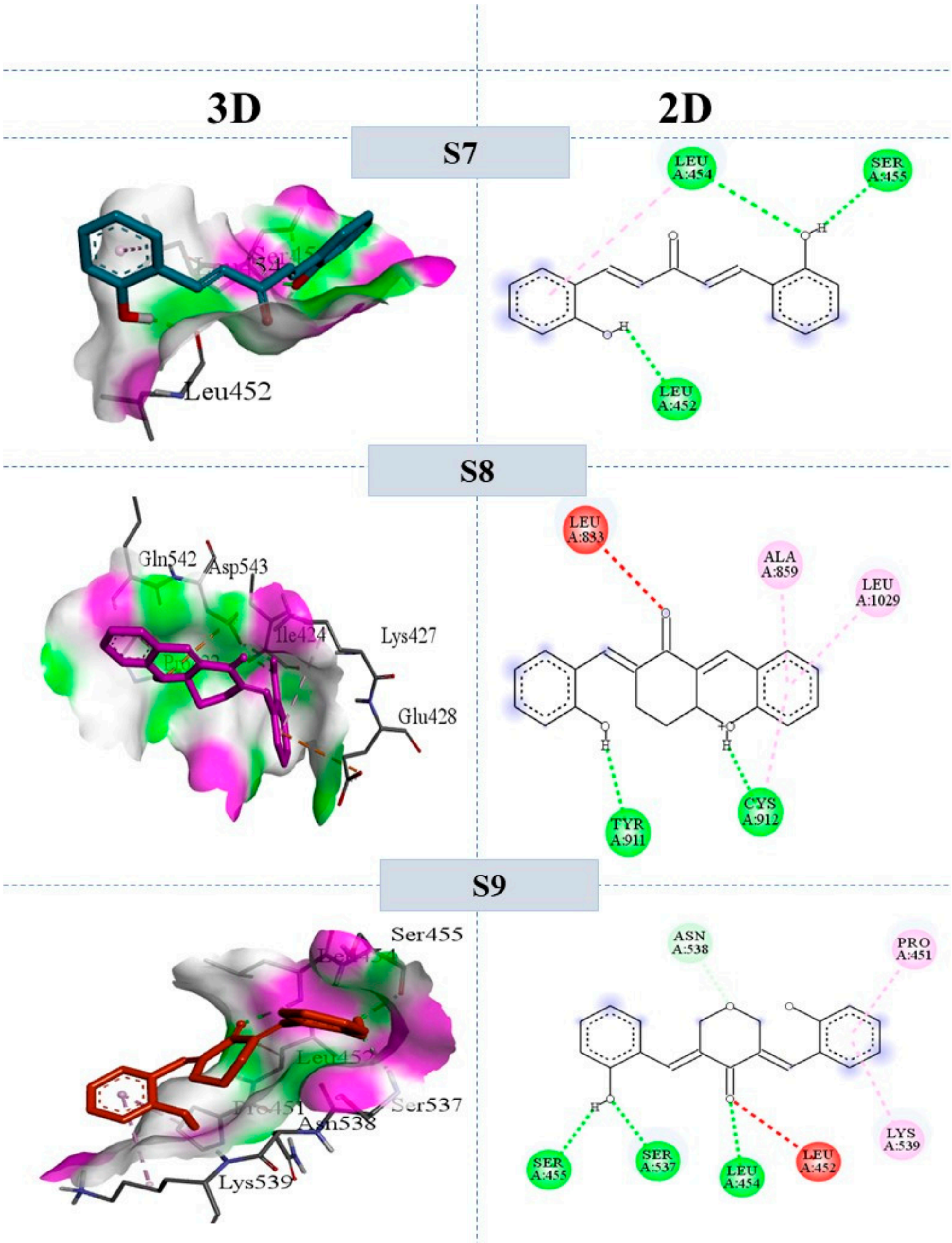

Figure S8. 3D and 2D interaction of S7, S8 and S9 within the active pocket of VEGFR-3 tyrosine kinase.

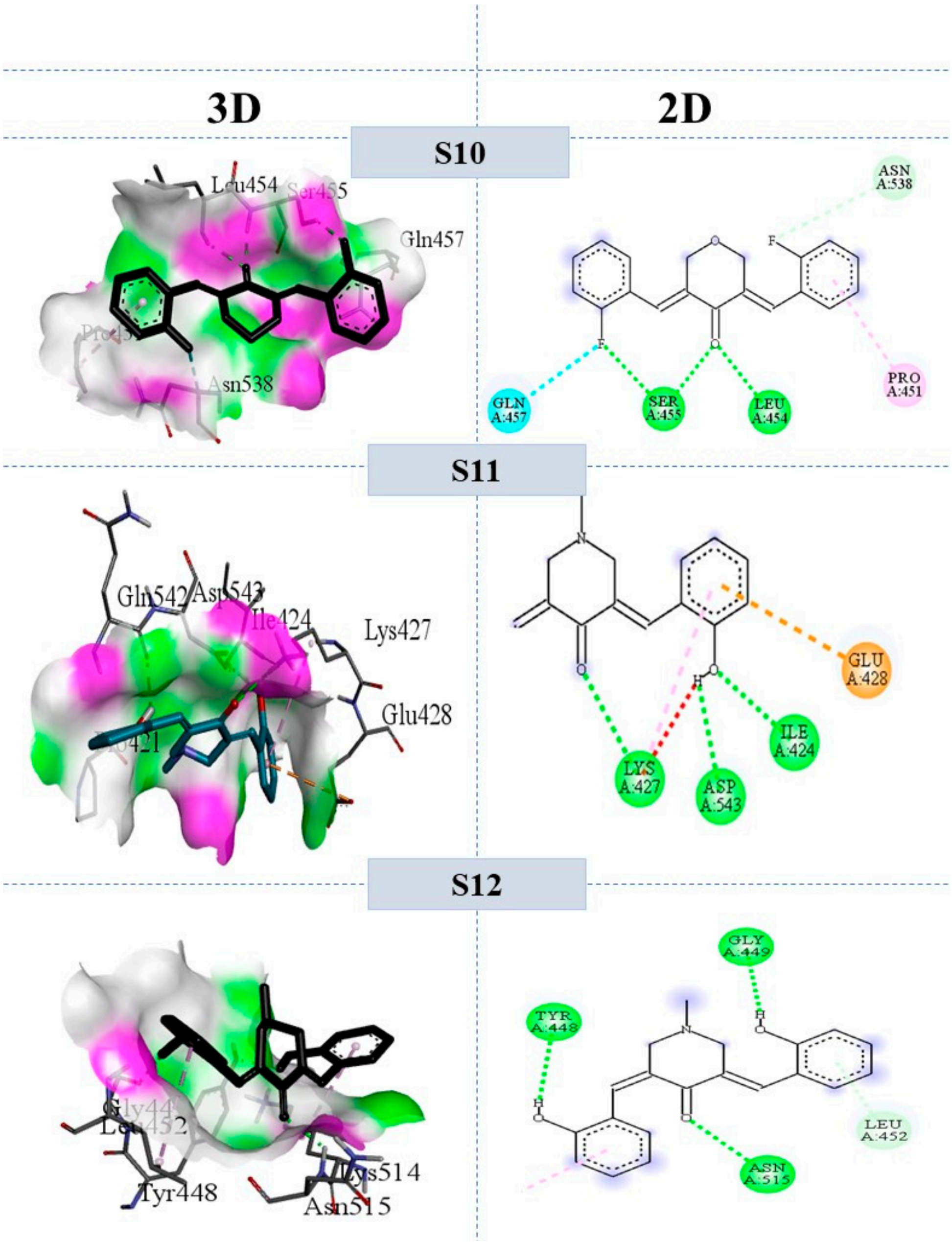

Figure S9. 3D and 2D interaction of S10, S11 and S12 within the active pocket of VEGFR-3 tyrosine kinase.

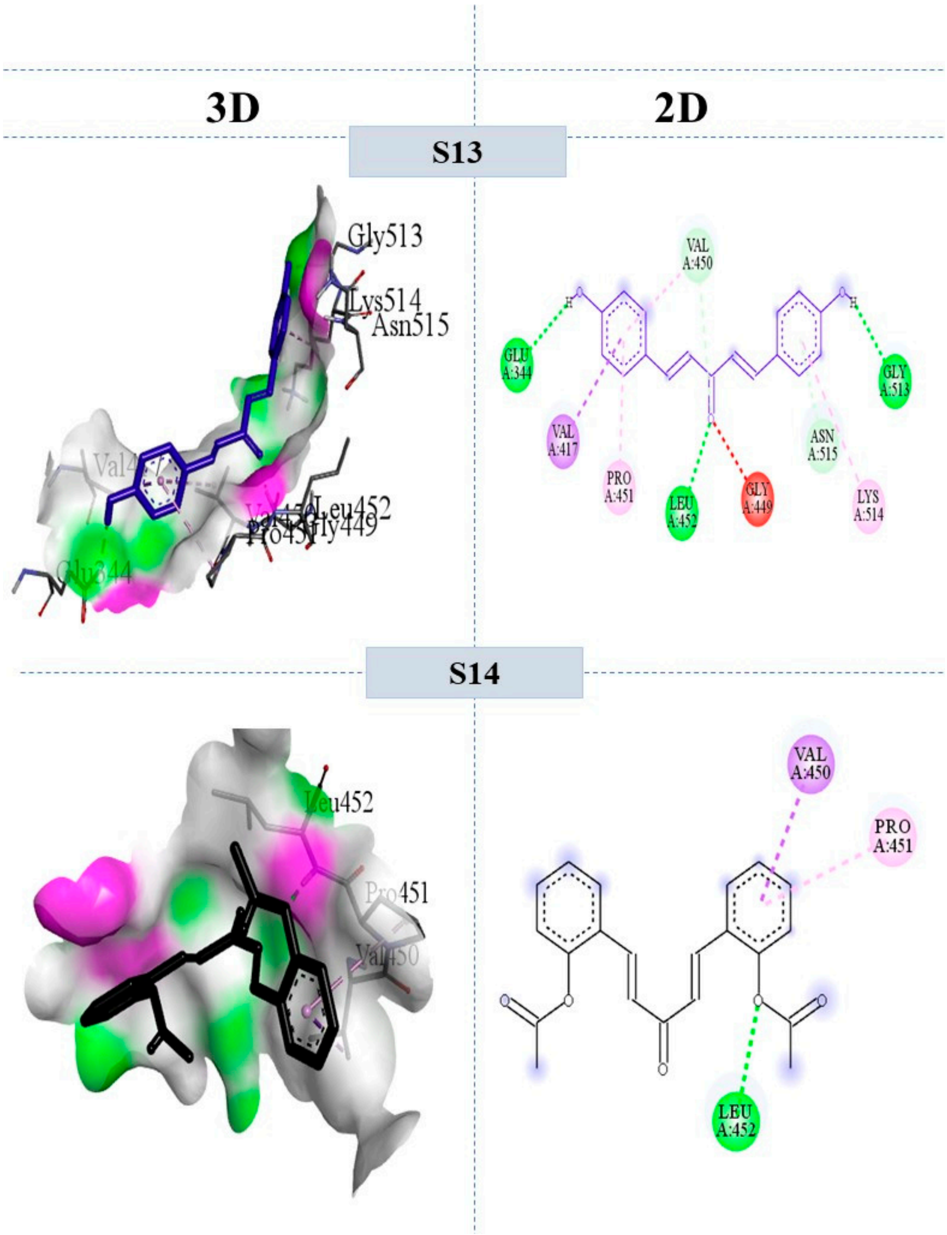

Figure S10. 3D and 2D interaction of S13 and S14 within the active pocket of VEGFR-3 tyrosine kinase.

Predicted Inhibitory constant (ki)

Table S1: Predicted inhibitory constant (ki)

Compound	ERBB tyrosine kinase Inhibition Constant (Ki)	VEGFR2 kinase Inhibition Constant (Ki)	VEGFR3 kinase Inhibition Constant (Ki)	VEGFR1 kinase Inhibition Constant (Ki)
S1	45 µM	86 µM	82.76 µM	550.7 nM
S2	22 µM	34 µM	38.37 µM	341 nM
S3	34 µM	345 µM	8.84 µM	8.74 µM
S4	232 µM	78 µM	219.78 µM	1.13 µM
S5	123 µM	234 µM	157.34 µM	1.50 µM
S6	33 µM	345 µM	370.46 µM	2.03 µM
S7	88 µM	76 µM	326.73 µM	2.1 µM
S8	96 µM	45 µM	5.63 µM	25.3 nM
S9	54 µM	23 µM	13.91 µM	48.6 nM
S10	53 µM	452 µM	130.25 µM	110.1 µM
S11	49.01nM	191.25 nM	8.15 µM	258.29 nM
S12	443 µM	53 µM	16.41 µM	1.03 µM
S13	331 µM	674 µM	366.04 µM	1.19 µM
S14	1.86 µM	199.15 nM	882.3 µM	457.5 nM
Irinotecan (reference)	73.05nM	70.99 nM		

References:

1. Tresaugues, L.; Roos, A.; Arrowsmith, C.H.; Berglund, H.; Bountra, C.; Collins, R.; Edwards, A.M.; Flodin, S.; Flores, A.; Graslund, S.; et al. Crystal structure of VEGFR1 in complex with N-(4-Chlorophenyl)-2-((pyridin-4-ylmethyl) amino) benzamide. The RCSB PDB. 2009.

2. Leppänen, V.M; Tvorogov, D.; Kisko, K.; Prota, A. E.; Jeltsch, M.; Anisimov, A.; Markovic-Mueller, S.; Stuttfeld, E.; Goldie, K.N.; Ballmer-Hofer, K.; et al. Structural and mechanistic

insights into VEGF receptor 3 ligand binding and activation. Proc. of the Natl. Acad. of Sci. 2013, 110(32), 12960-12965.
